# Supplementary figures and images for: Microbiota derived d-malate inhibits skeletal muscle growth and angiogenesis during aging via acetylation of Cyclin A
Source: EMBO Rep. 2024 Jan 22;25(2):9. doi: 10.1038/s44319-023-00028-y (PMC10897302; doi:10.1038/s44319-023-00028-y)

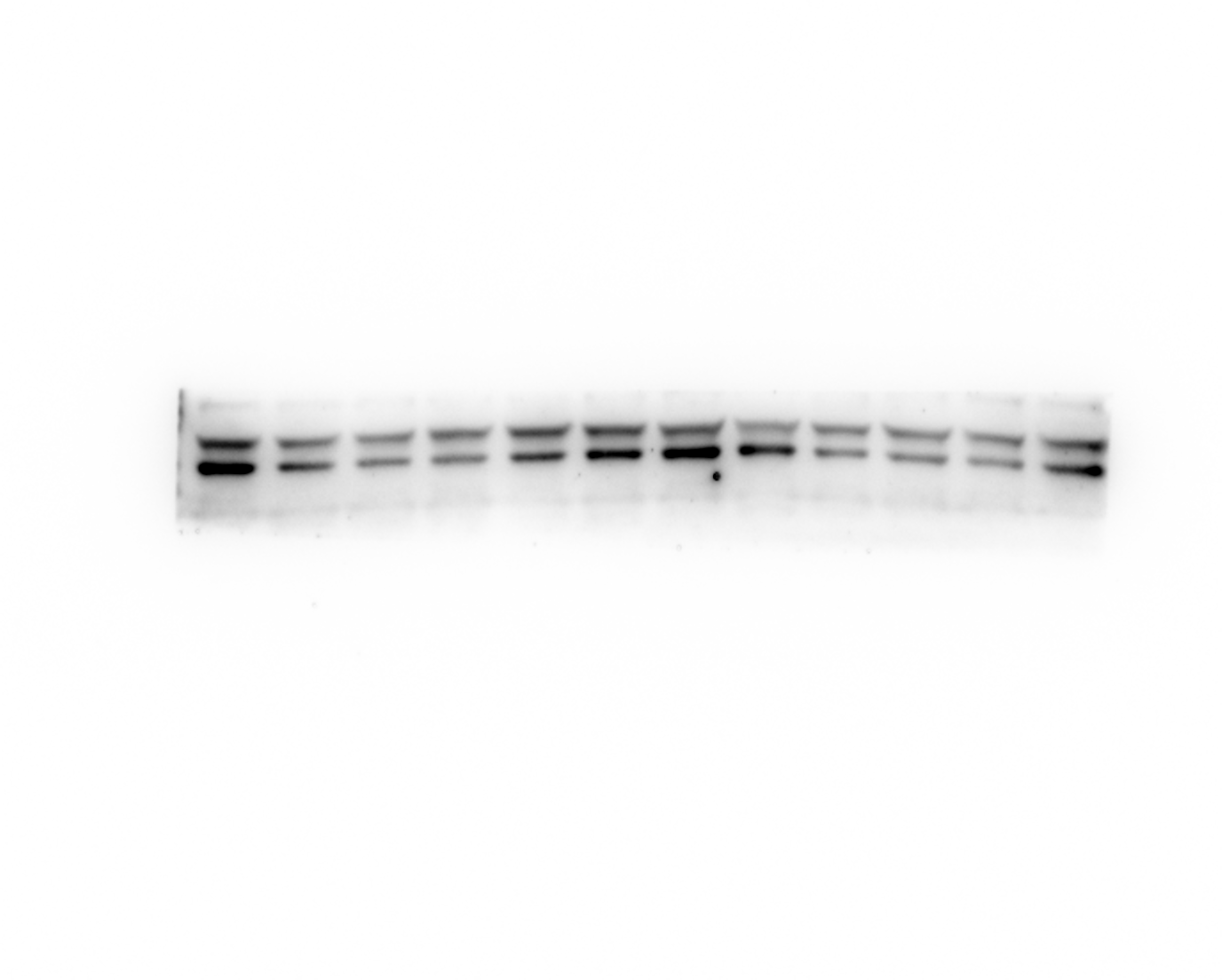

Supplement: Supplementary file 4 — Source Data Fig. 3 [file 44319_2023_28_MOESM4_ESM.zip › EMBOR-2023-57167V1_SourceDataForFigure 3/Fig 3M GAPDH.Tif]

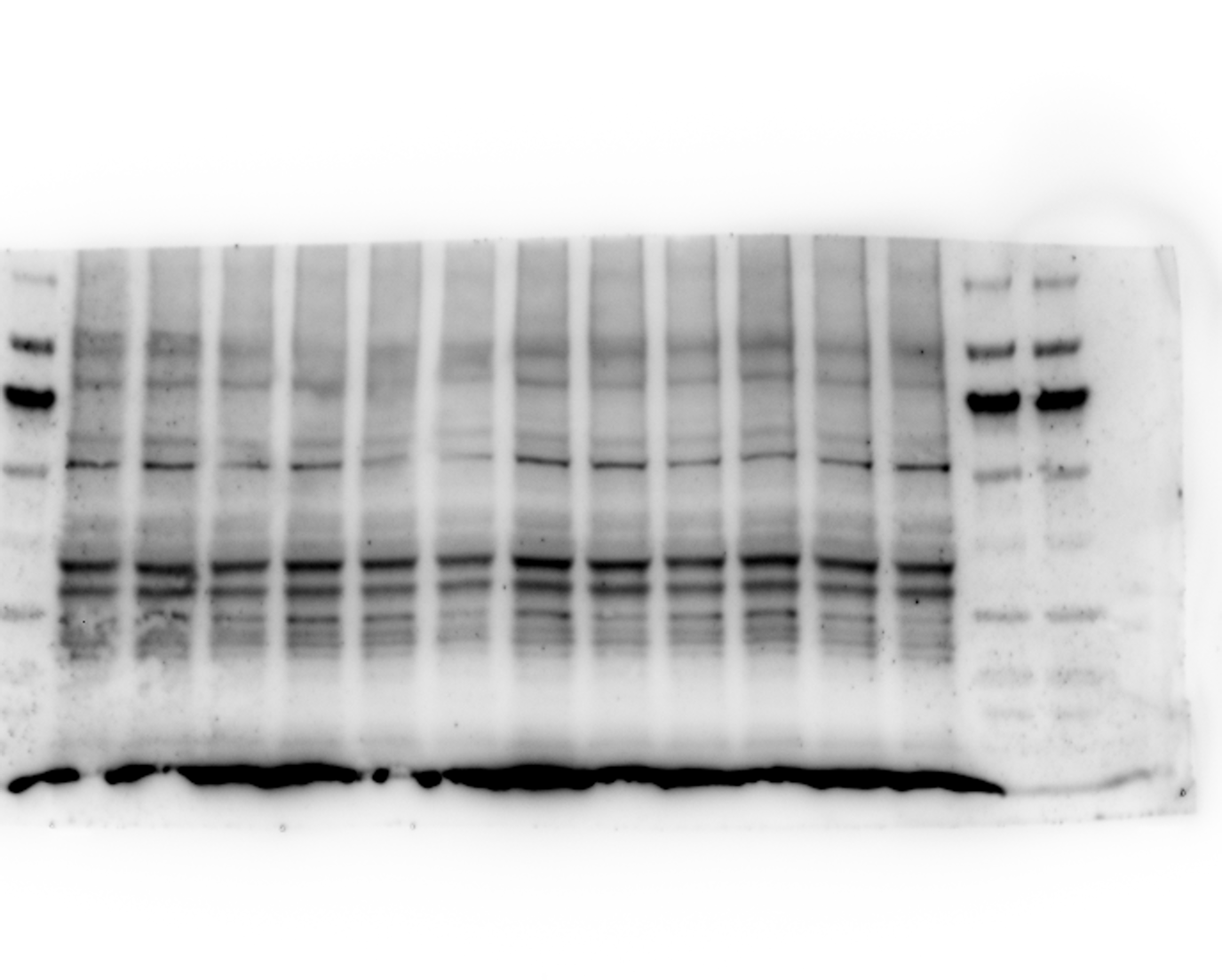

Supplement: Supplementary file 4 — Source Data Fig. 3 [file 44319_2023_28_MOESM4_ESM.zip › EMBOR-2023-57167V1_SourceDataForFigure 3/Fig 3M Ubiquitin.Tif]

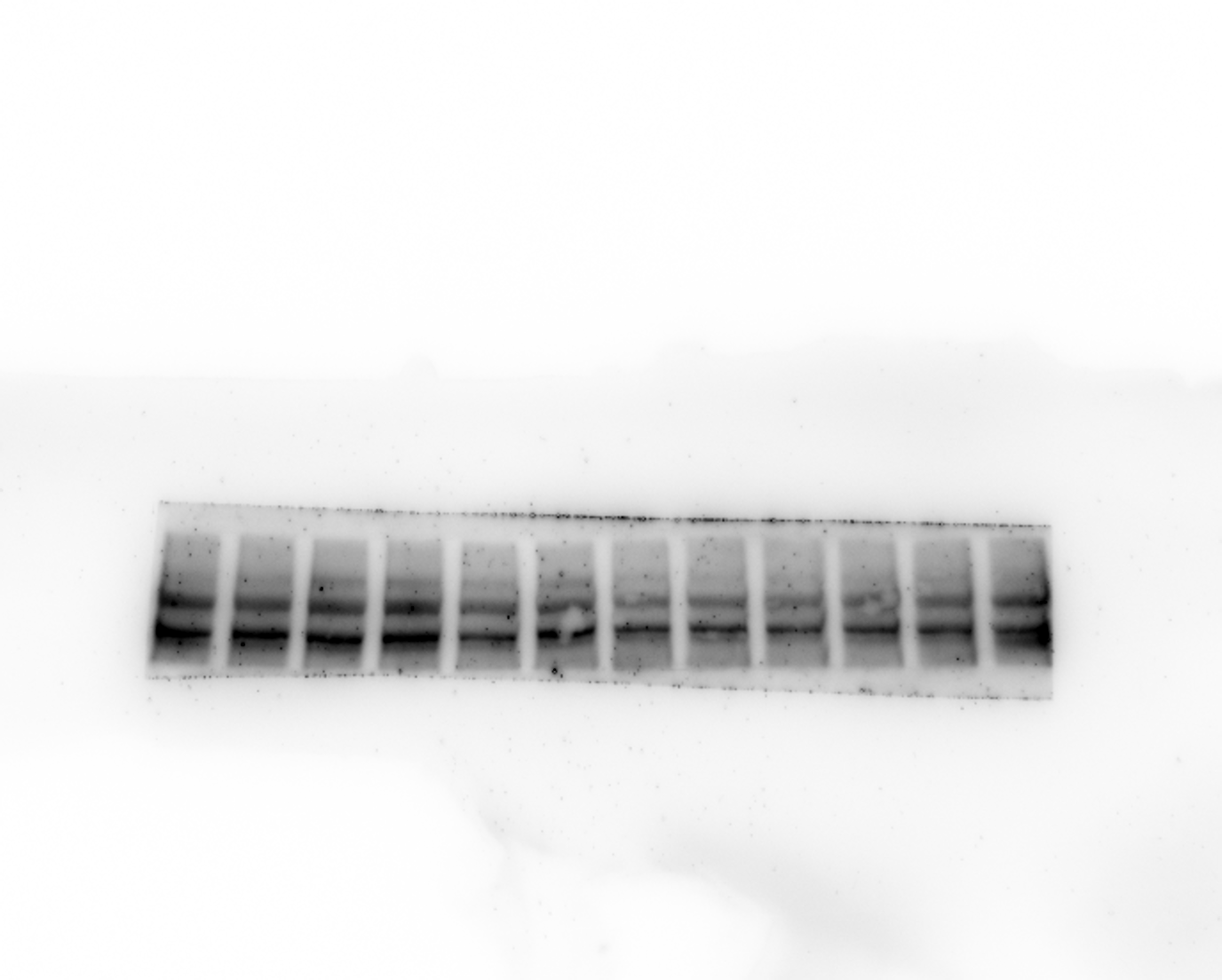

Supplement: Supplementary file 5 — Source Data Fig. 4 [file 44319_2023_28_MOESM5_ESM.zip › EMBOR-2023-57167V1_SourceDataForFigure 4/Fig4E-4H/HIF2A.Tif]

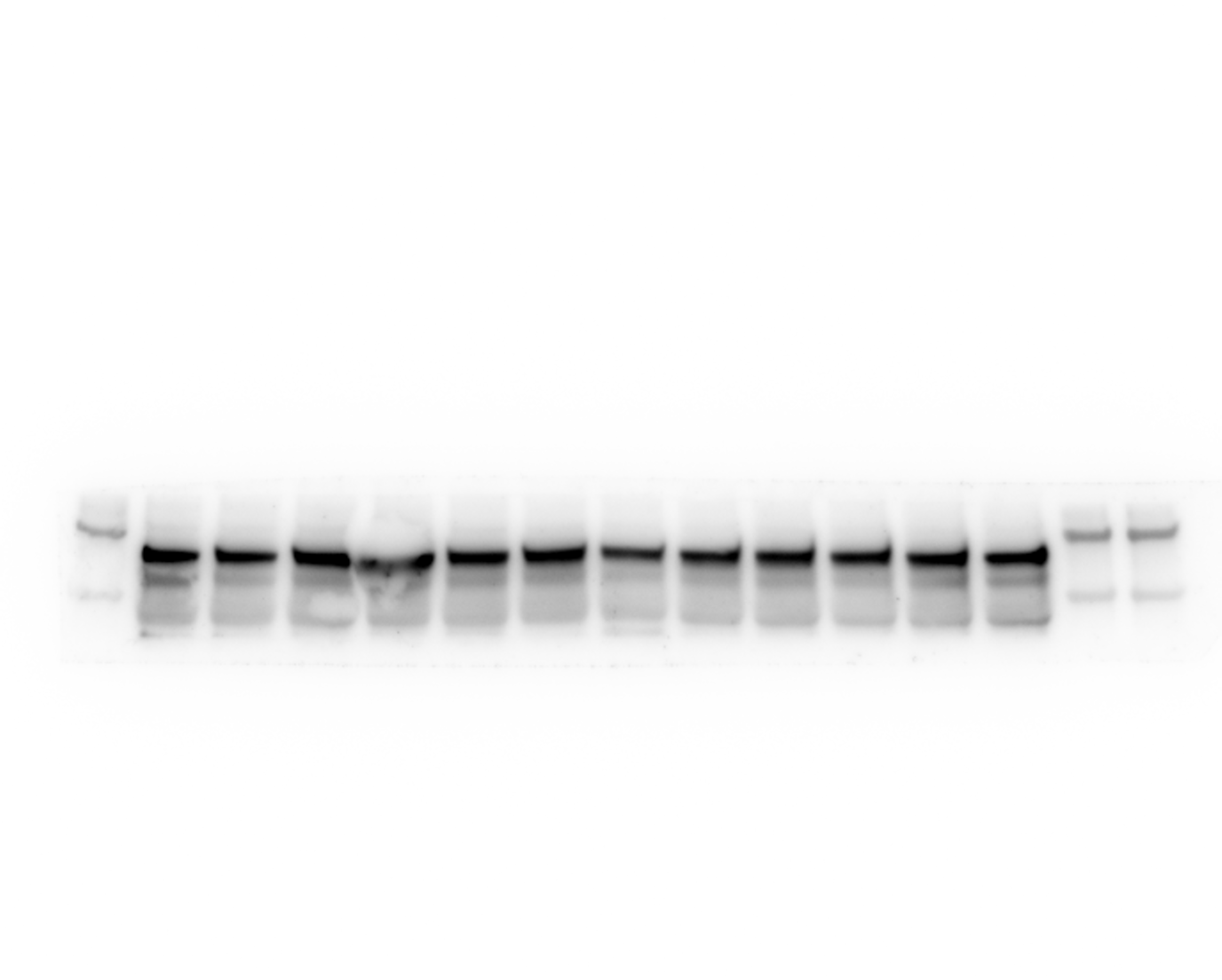

Supplement: Supplementary file 5 — Source Data Fig. 4 [file 44319_2023_28_MOESM5_ESM.zip › EMBOR-2023-57167V1_SourceDataForFigure 4/Fig4E-4H/TUBULIN-1.Tif]

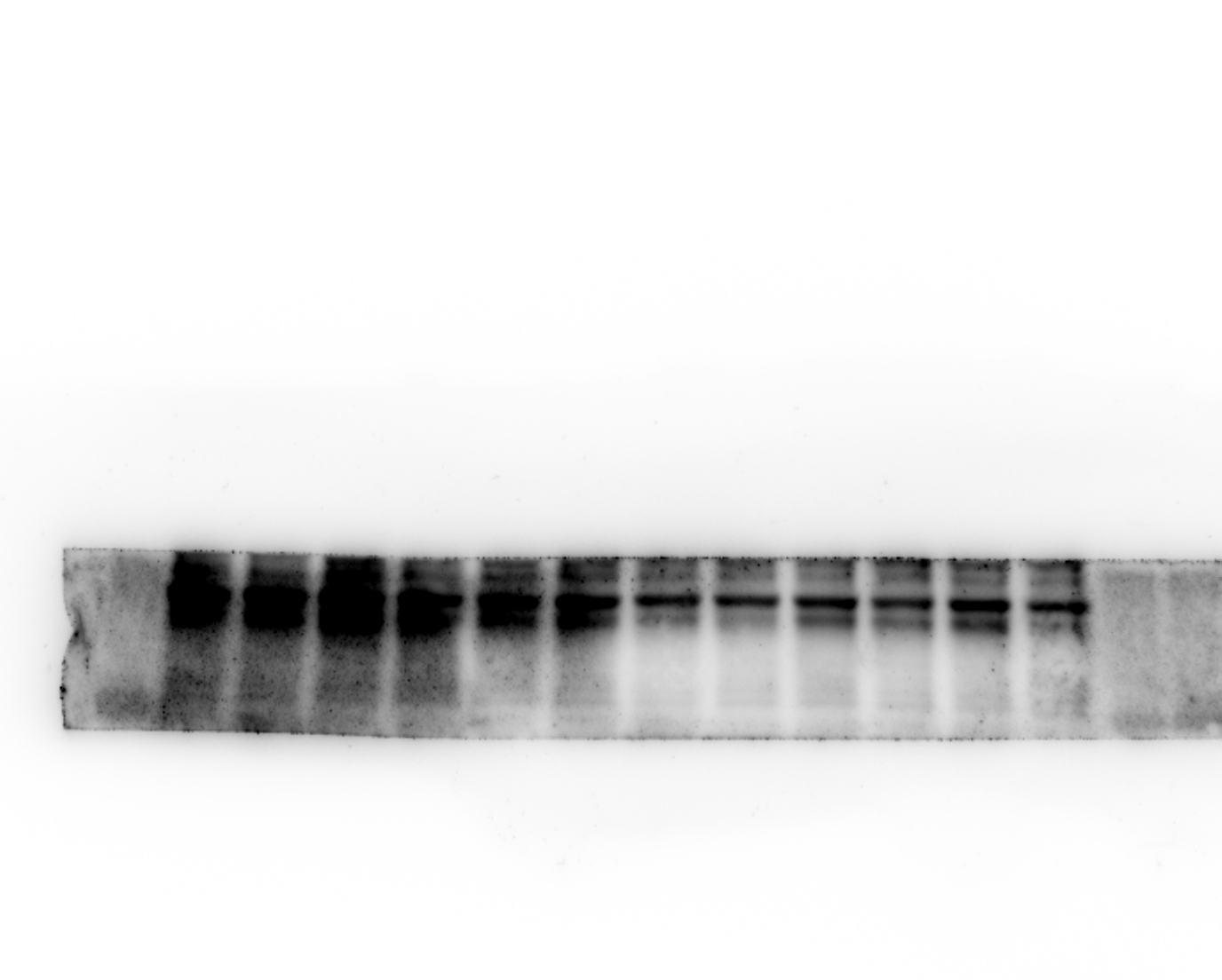

Supplement: Supplementary file 5 — Source Data Fig. 4 [file 44319_2023_28_MOESM5_ESM.zip › EMBOR-2023-57167V1_SourceDataForFigure 4/Fig4E-4H/VEGFB.Tif]

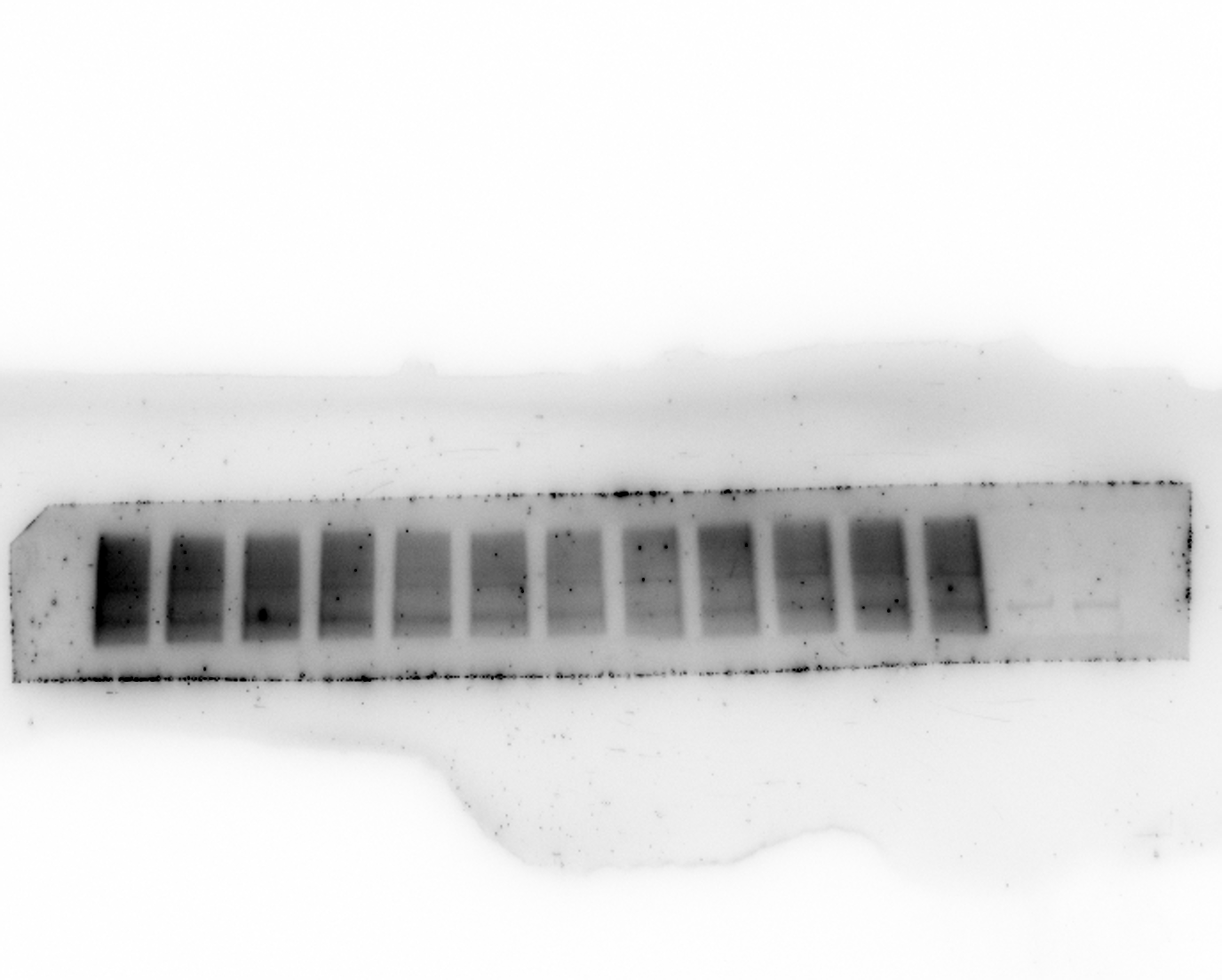

Supplement: Supplementary file 5 — Source Data Fig. 4 [file 44319_2023_28_MOESM5_ESM.zip › EMBOR-2023-57167V1_SourceDataForFigure 4/Fig4E-4H/VEGFR2.Tif]

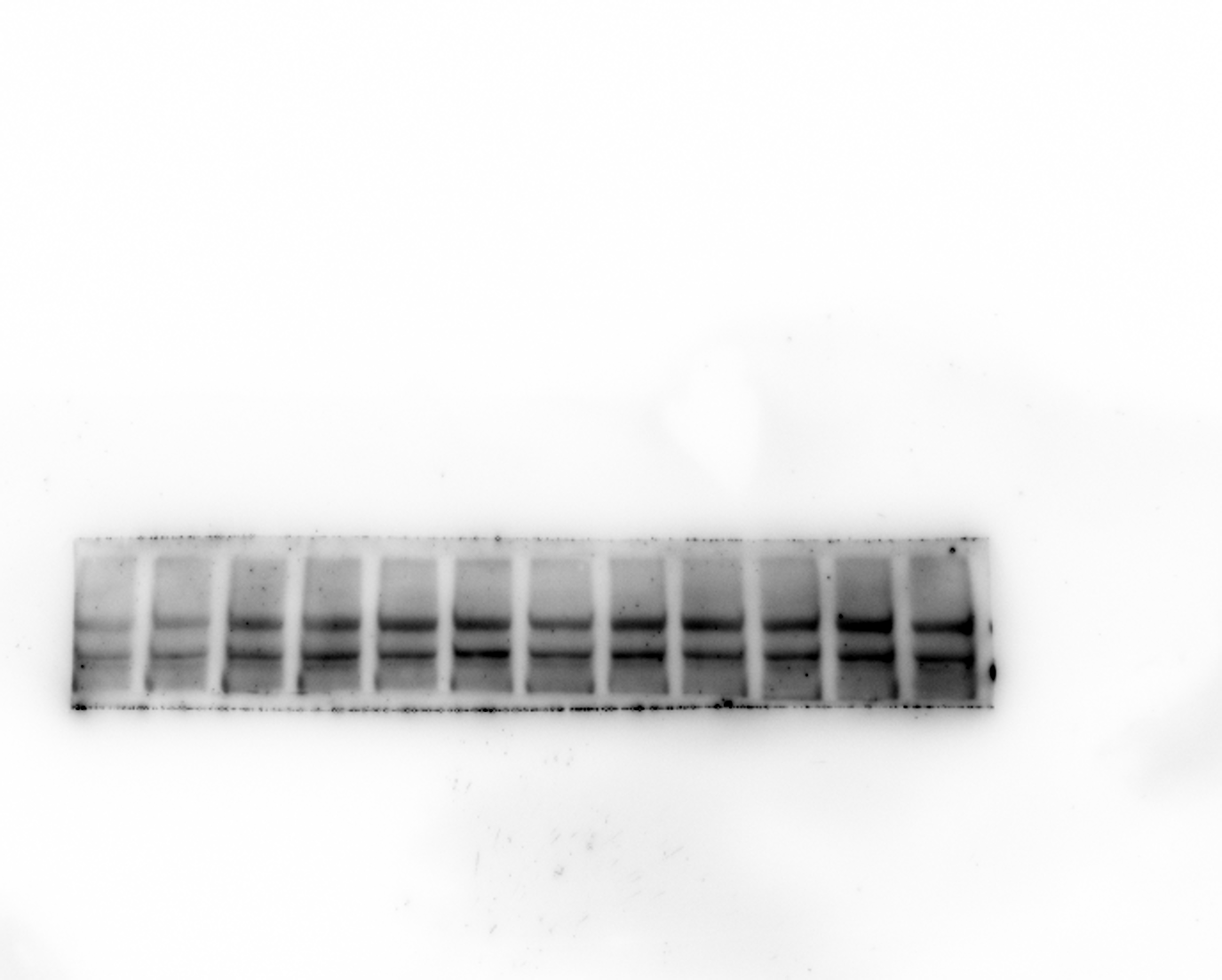

Supplement: Supplementary file 5 — Source Data Fig. 4 [file 44319_2023_28_MOESM5_ESM.zip › EMBOR-2023-57167V1_SourceDataForFigure 4/Fig4I-4L/HIF1A.Tif]

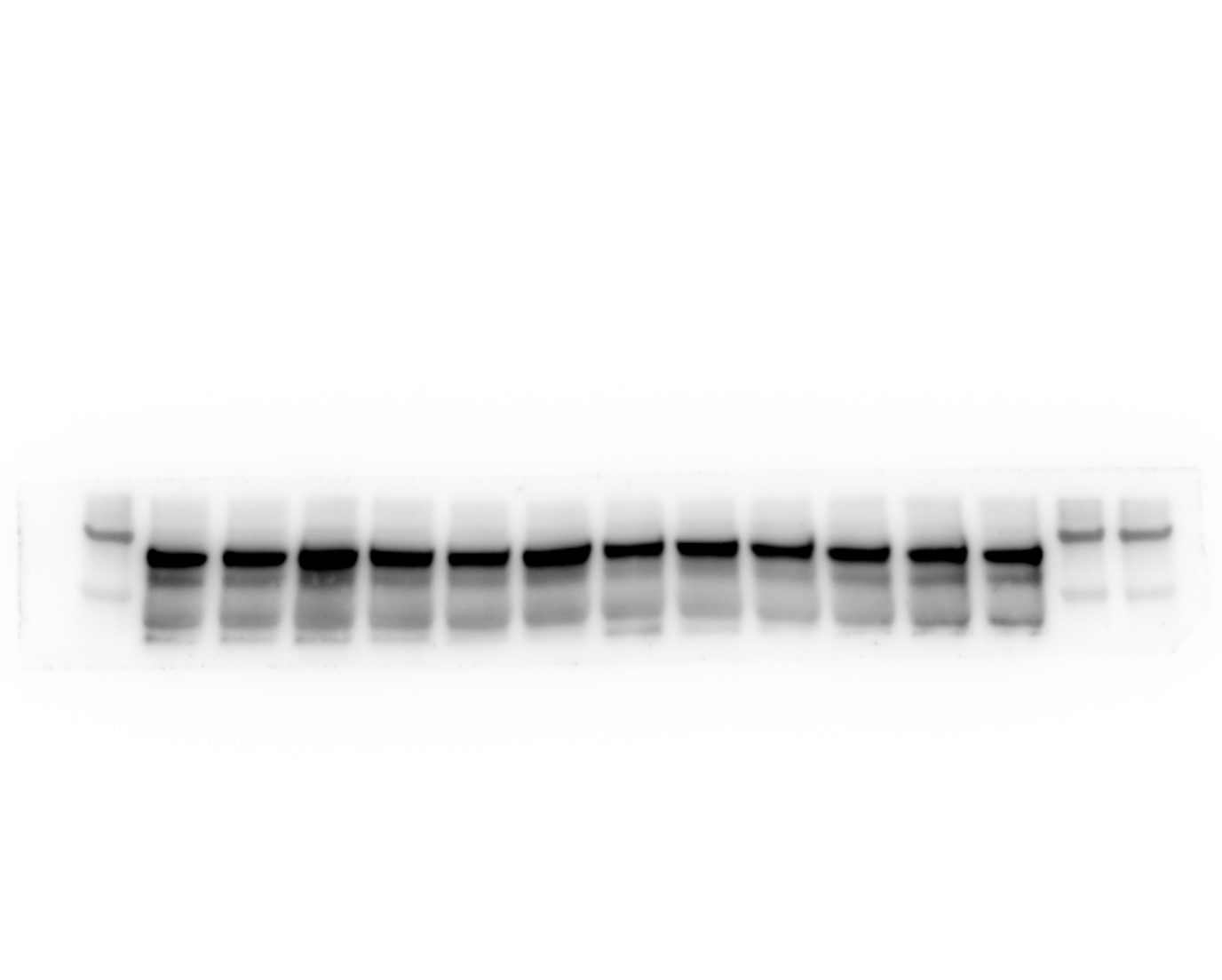

Supplement: Supplementary file 5 — Source Data Fig. 4 [file 44319_2023_28_MOESM5_ESM.zip › EMBOR-2023-57167V1_SourceDataForFigure 4/Fig4I-4L/TUBULIN-2.Tif]

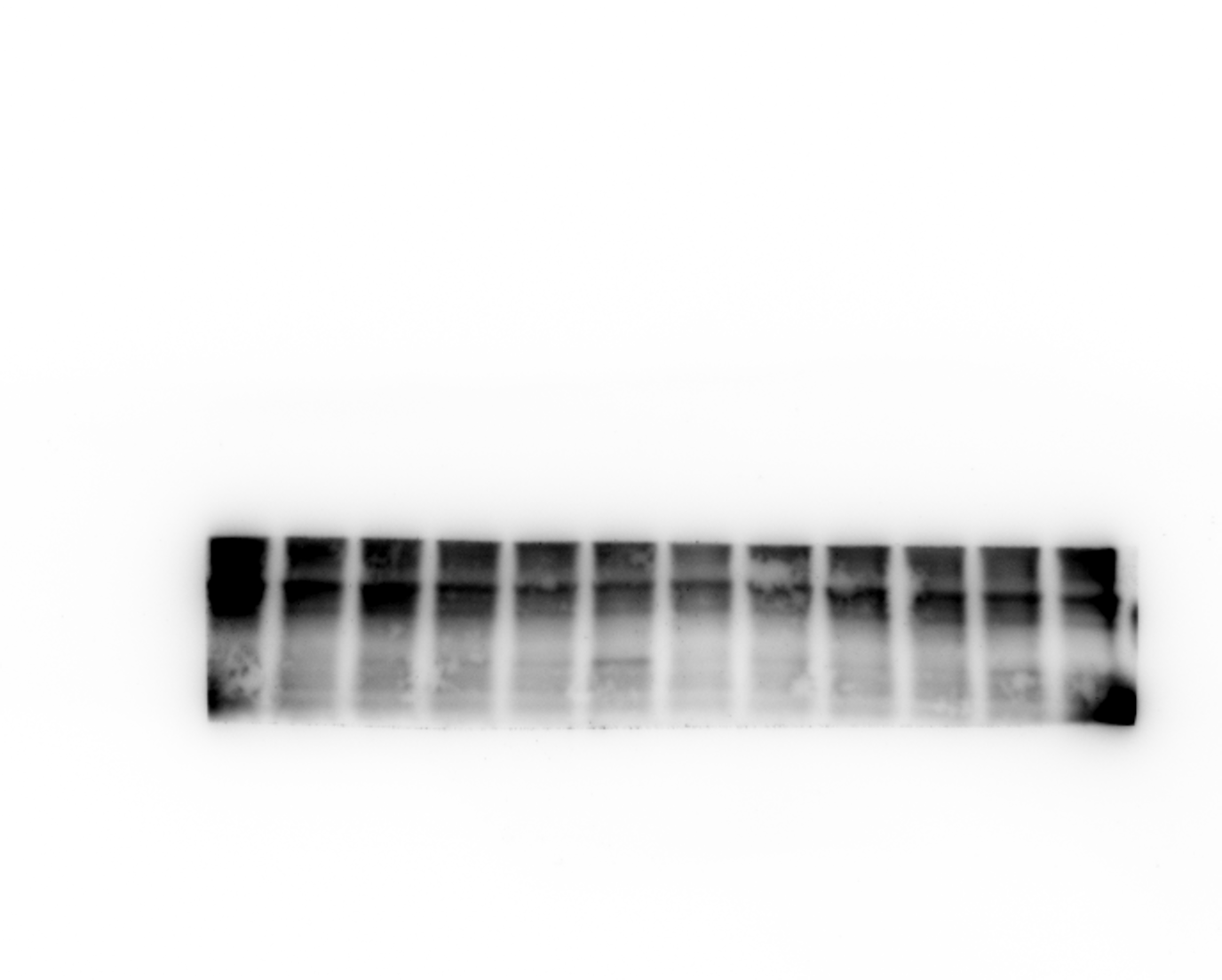

Supplement: Supplementary file 5 — Source Data Fig. 4 [file 44319_2023_28_MOESM5_ESM.zip › EMBOR-2023-57167V1_SourceDataForFigure 4/Fig4I-4L/VEGFA.Tif]

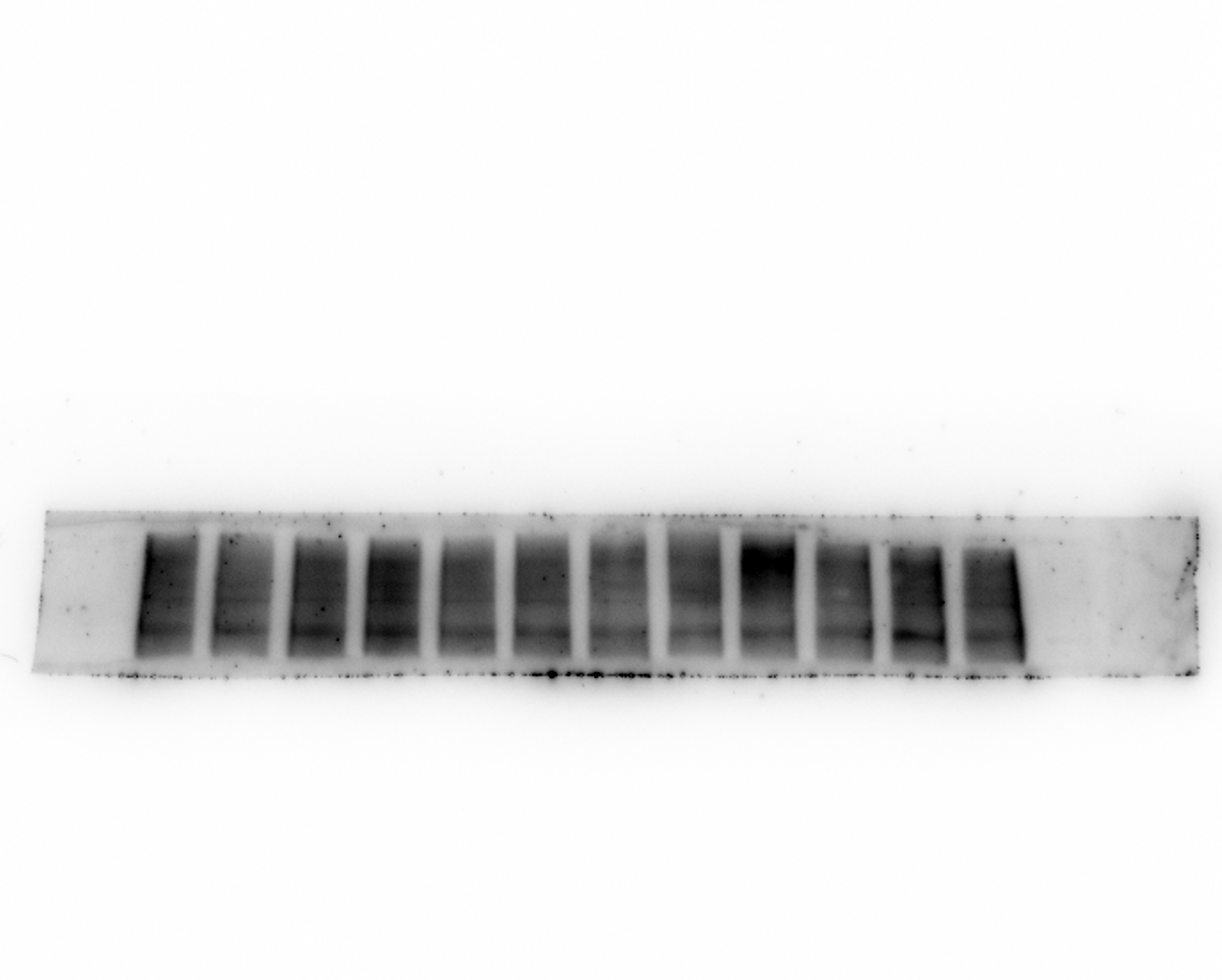

Supplement: Supplementary file 5 — Source Data Fig. 4 [file 44319_2023_28_MOESM5_ESM.zip › EMBOR-2023-57167V1_SourceDataForFigure 4/Fig4I-4L/VEGFR1.Tif]

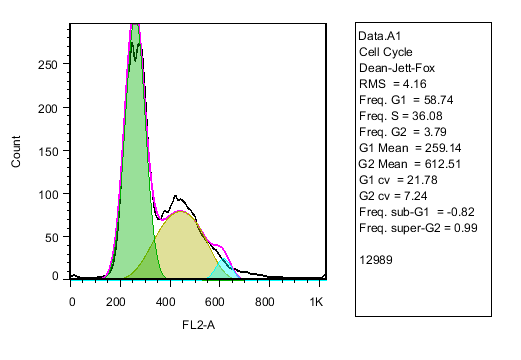

Supplement: Supplementary file 6 — Source Data Fig. 5 [file 44319_2023_28_MOESM6_ESM.zip › EMBOR-2023-57167V1_SourceDataForFigure 5/Fig 5G-H source data of PVEC/A1.png]

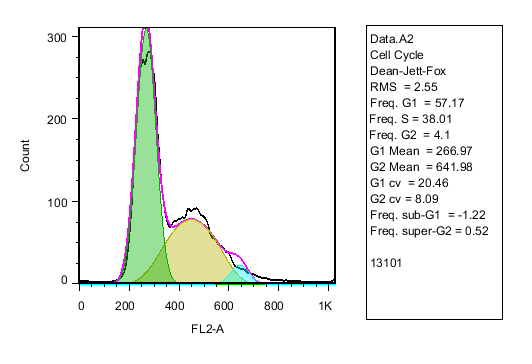

Supplement: Supplementary file 6 — Source Data Fig. 5 [file 44319_2023_28_MOESM6_ESM.zip › EMBOR-2023-57167V1_SourceDataForFigure 5/Fig 5G-H source data of PVEC/A2.png]

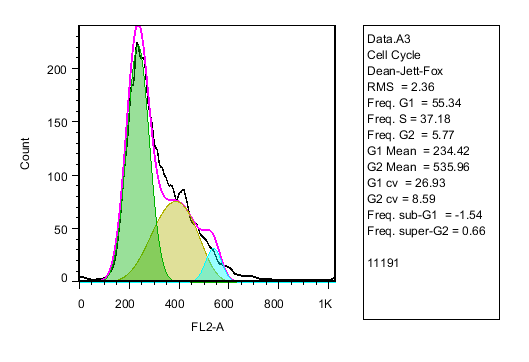

Supplement: Supplementary file 6 — Source Data Fig. 5 [file 44319_2023_28_MOESM6_ESM.zip › EMBOR-2023-57167V1_SourceDataForFigure 5/Fig 5G-H source data of PVEC/A3.png]

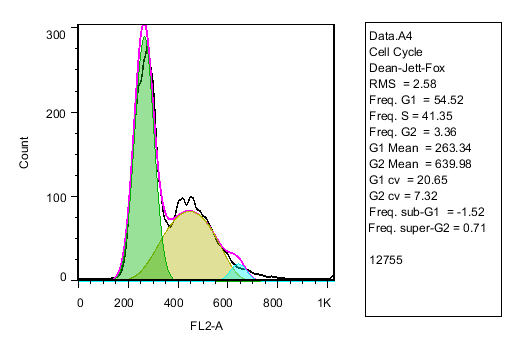

Supplement: Supplementary file 6 — Source Data Fig. 5 [file 44319_2023_28_MOESM6_ESM.zip › EMBOR-2023-57167V1_SourceDataForFigure 5/Fig 5G-H source data of PVEC/A4.png]

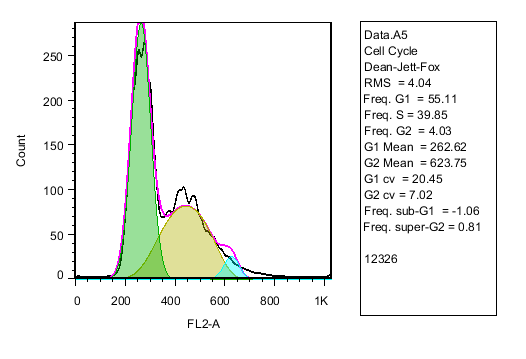

Supplement: Supplementary file 6 — Source Data Fig. 5 [file 44319_2023_28_MOESM6_ESM.zip › EMBOR-2023-57167V1_SourceDataForFigure 5/Fig 5G-H source data of PVEC/A5.png]

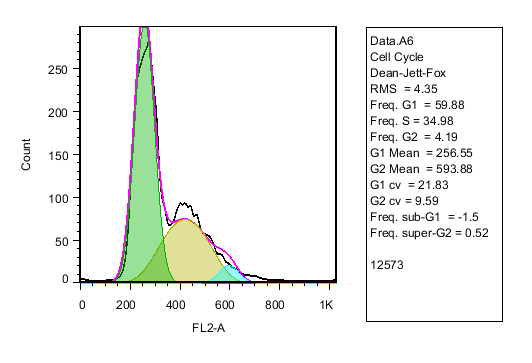

Supplement: Supplementary file 6 — Source Data Fig. 5 [file 44319_2023_28_MOESM6_ESM.zip › EMBOR-2023-57167V1_SourceDataForFigure 5/Fig 5G-H source data of PVEC/A6.png]

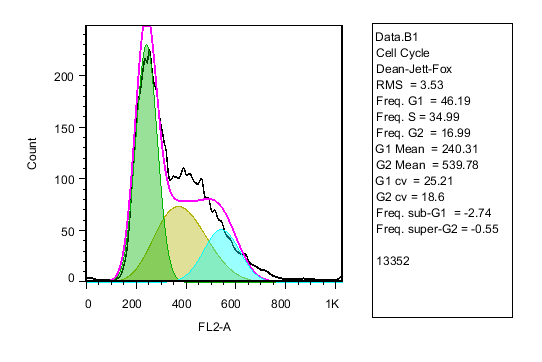

Supplement: Supplementary file 6 — Source Data Fig. 5 [file 44319_2023_28_MOESM6_ESM.zip › EMBOR-2023-57167V1_SourceDataForFigure 5/Fig 5G-H source data of PVEC/B1.png]

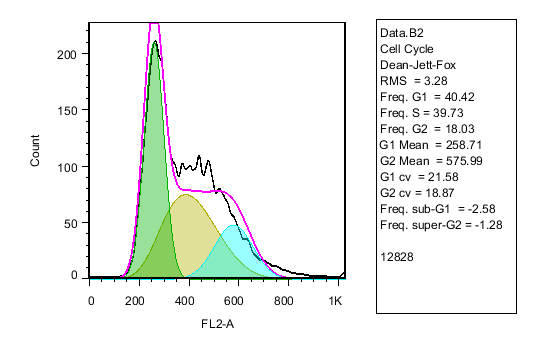

Supplement: Supplementary file 6 — Source Data Fig. 5 [file 44319_2023_28_MOESM6_ESM.zip › EMBOR-2023-57167V1_SourceDataForFigure 5/Fig 5G-H source data of PVEC/B2.png]

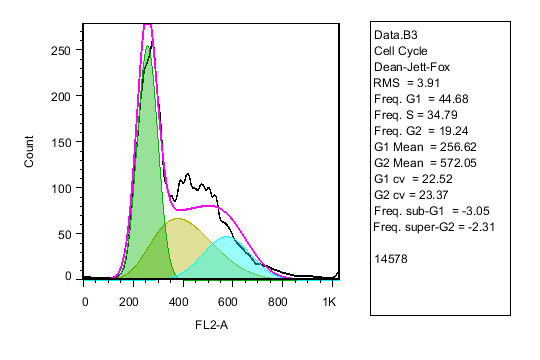

Supplement: Supplementary file 6 — Source Data Fig. 5 [file 44319_2023_28_MOESM6_ESM.zip › EMBOR-2023-57167V1_SourceDataForFigure 5/Fig 5G-H source data of PVEC/B3.png]

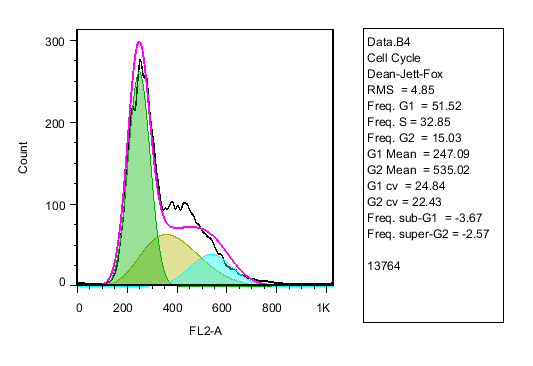

Supplement: Supplementary file 6 — Source Data Fig. 5 [file 44319_2023_28_MOESM6_ESM.zip › EMBOR-2023-57167V1_SourceDataForFigure 5/Fig 5G-H source data of PVEC/B4.png]

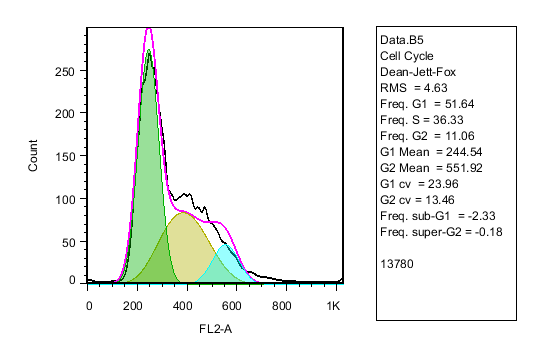

Supplement: Supplementary file 6 — Source Data Fig. 5 [file 44319_2023_28_MOESM6_ESM.zip › EMBOR-2023-57167V1_SourceDataForFigure 5/Fig 5G-H source data of PVEC/B5.png]

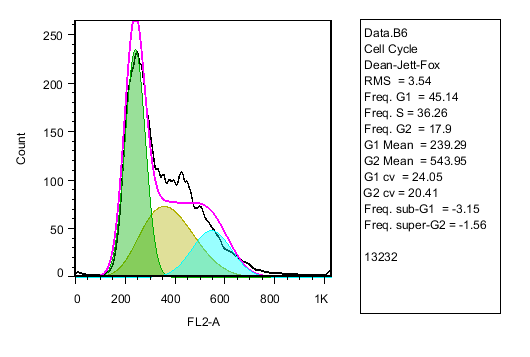

Supplement: Supplementary file 6 — Source Data Fig. 5 [file 44319_2023_28_MOESM6_ESM.zip › EMBOR-2023-57167V1_SourceDataForFigure 5/Fig 5G-H source data of PVEC/B6.png]

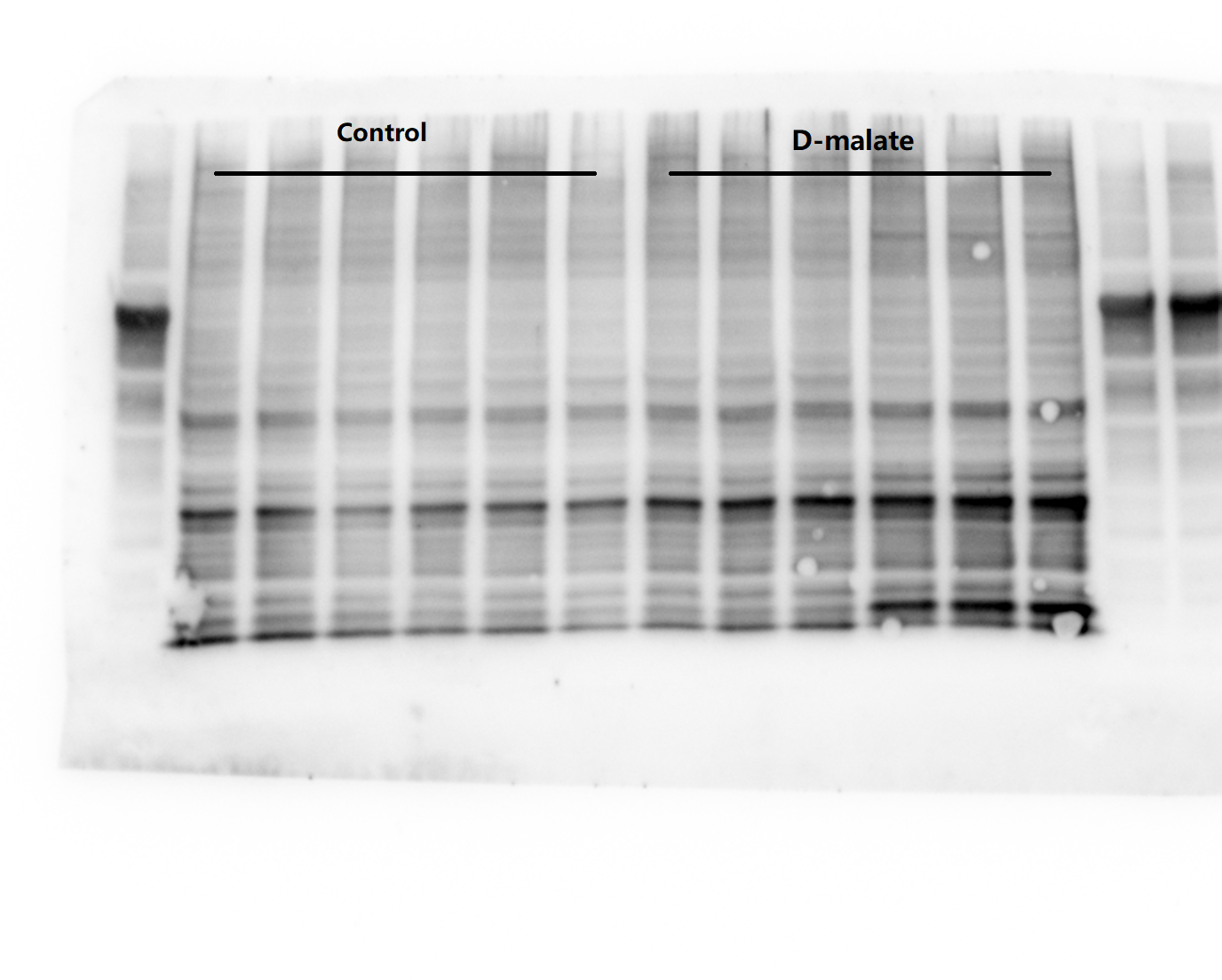

Supplement: Supplementary file 8 — Source Data Fig. 7 [file 44319_2023_28_MOESM8_ESM.zip › EMBOR-2023-57167V1_SourceDataForFigure 7/Fig 7E Acetylatio of total protein in PVEC (1).Tif]

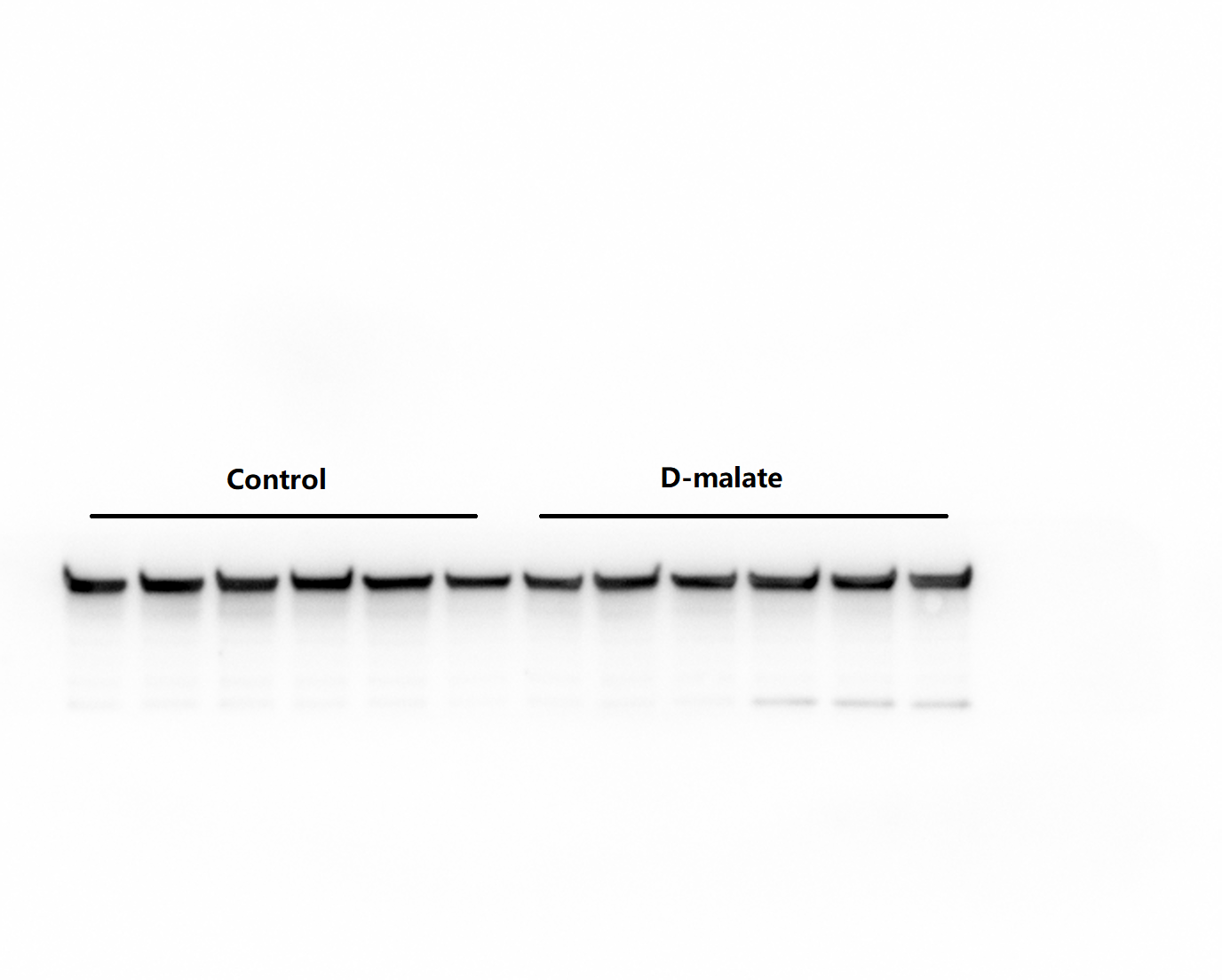

Supplement: Supplementary file 8 — Source Data Fig. 7 [file 44319_2023_28_MOESM8_ESM.zip › EMBOR-2023-57167V1_SourceDataForFigure 7/Fig 7E Tubulin.Tif]

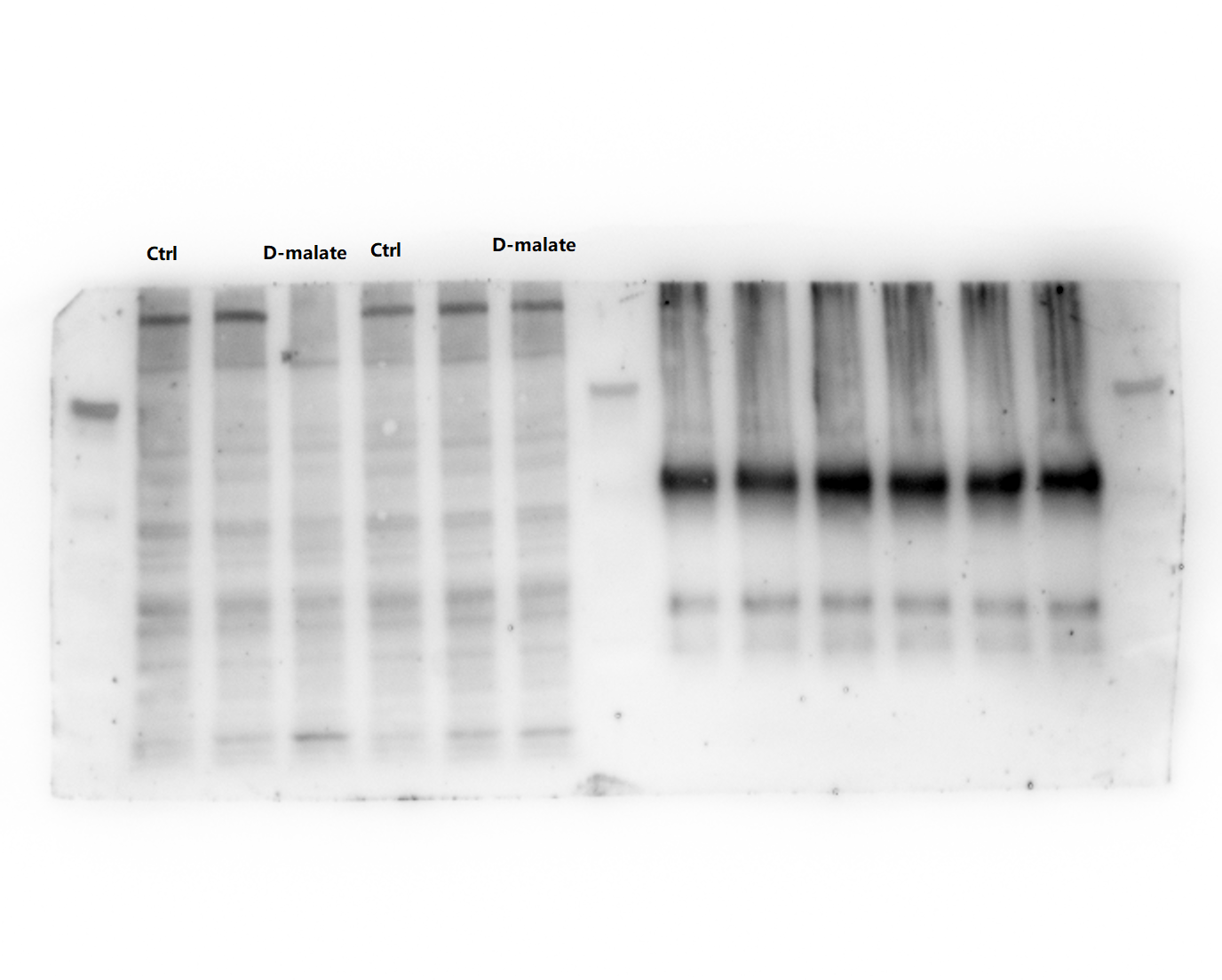

Supplement: Supplementary file 8 — Source Data Fig. 7 [file 44319_2023_28_MOESM8_ESM.zip › EMBOR-2023-57167V1_SourceDataForFigure 7/Fig 7G Ace-Cyclin A2 (replication).Tif]

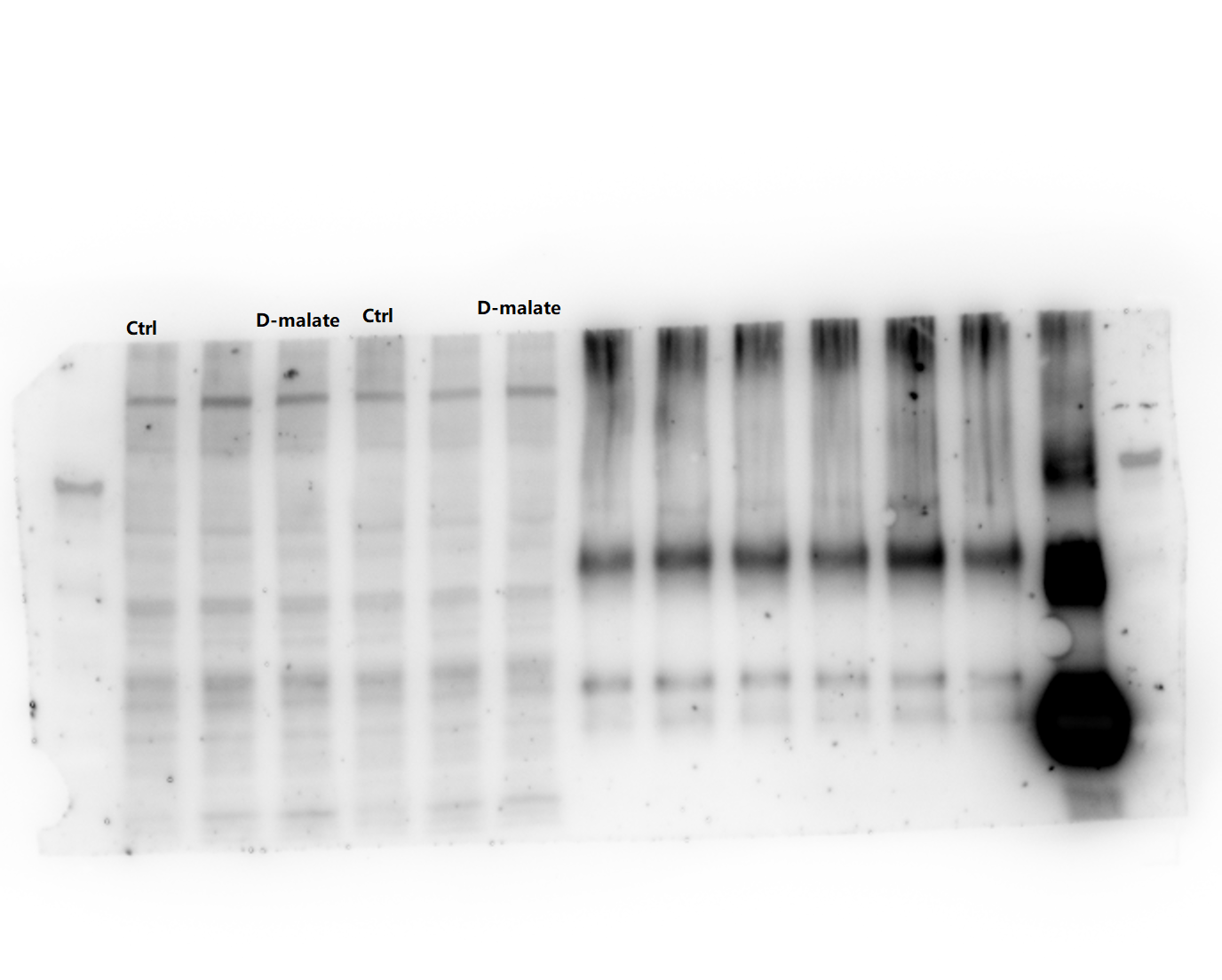

Supplement: Supplementary file 8 — Source Data Fig. 7 [file 44319_2023_28_MOESM8_ESM.zip › EMBOR-2023-57167V1_SourceDataForFigure 7/Fig 7G Ace-Cyclin A2.Tif]

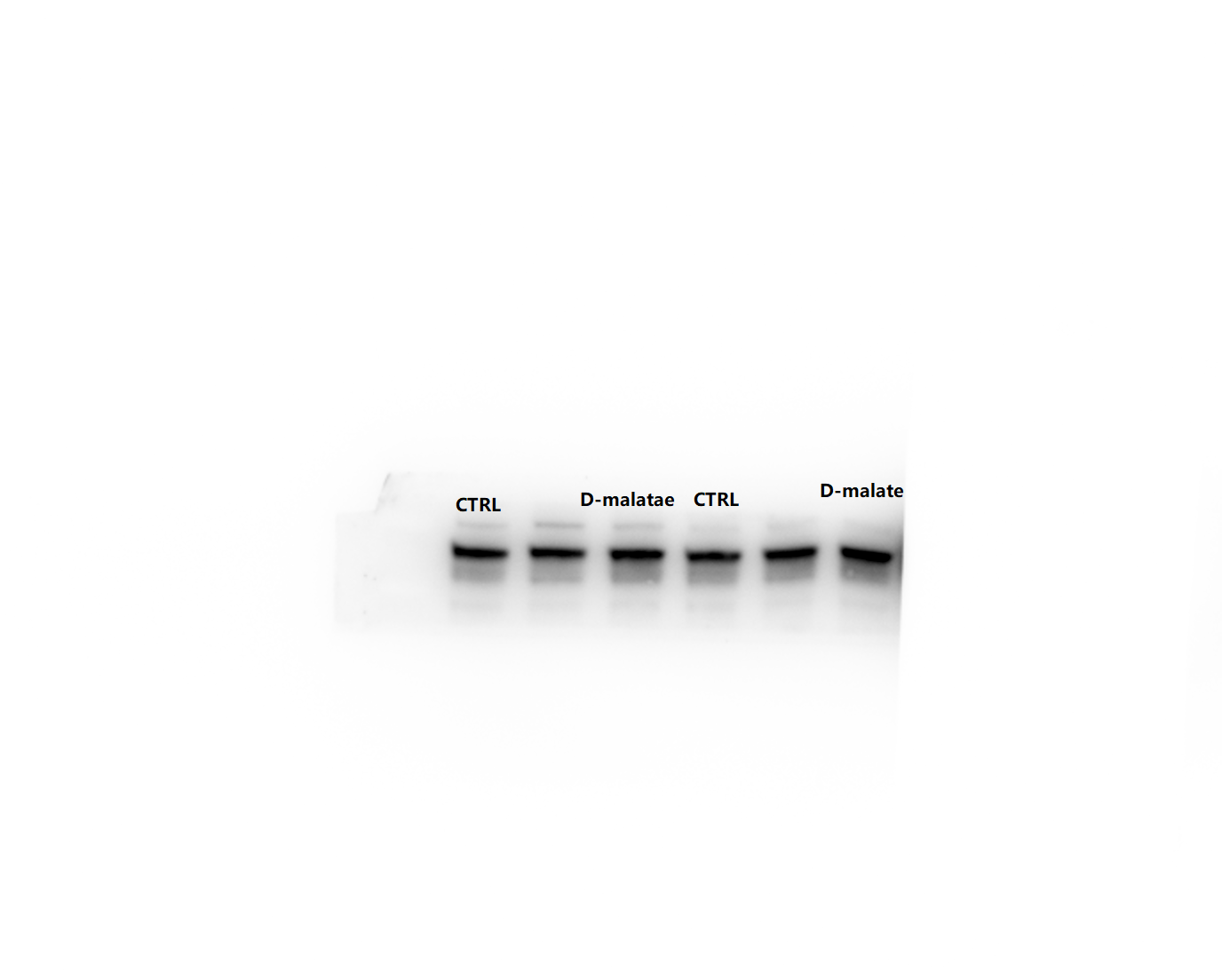

Supplement: Supplementary file 8 — Source Data Fig. 7 [file 44319_2023_28_MOESM8_ESM.zip › EMBOR-2023-57167V1_SourceDataForFigure 7/Fig 7G Input CyclinA.Tif]

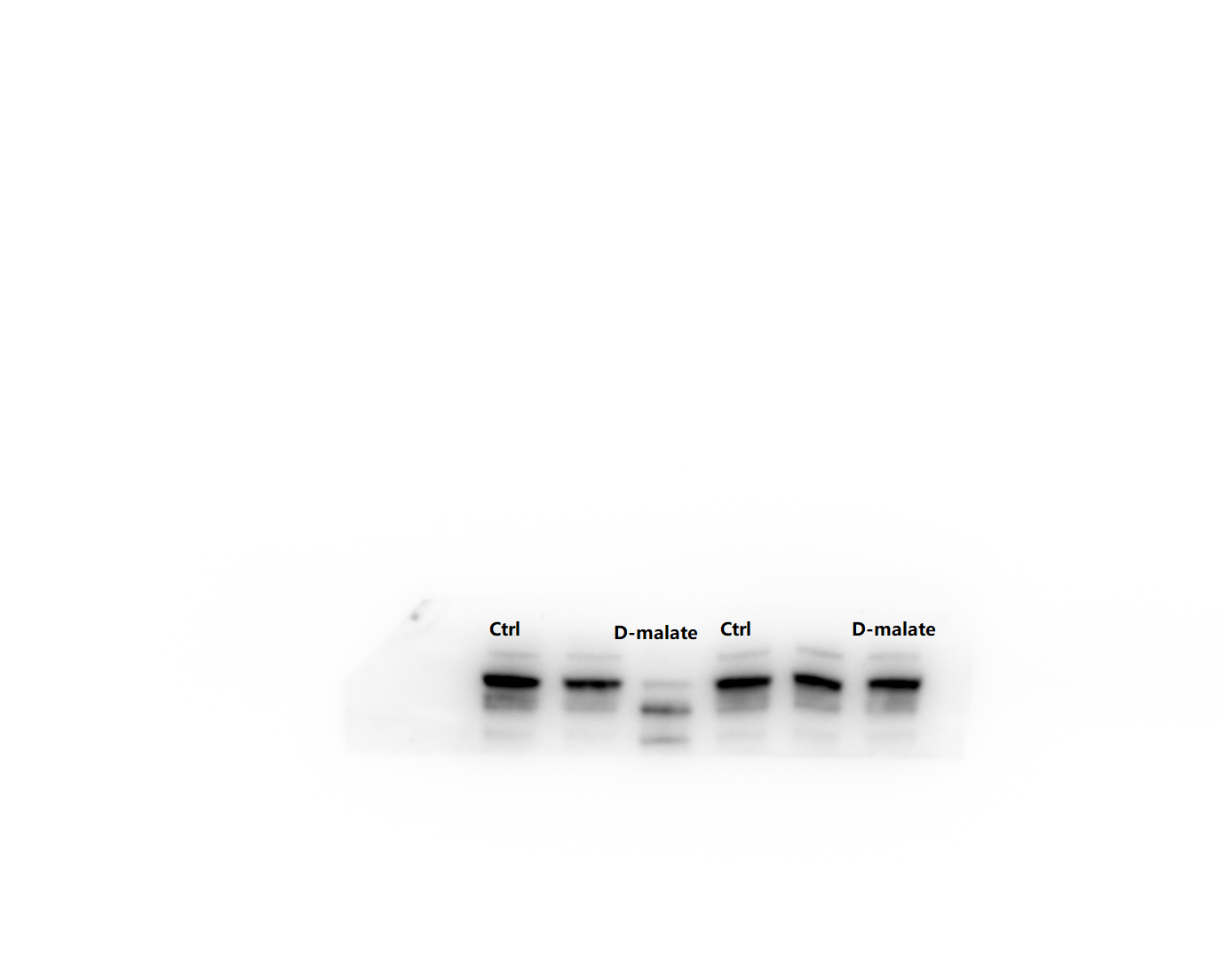

Supplement: Supplementary file 8 — Source Data Fig. 7 [file 44319_2023_28_MOESM8_ESM.zip › EMBOR-2023-57167V1_SourceDataForFigure 7/Fig 7G Input CyclinA(replication).Tif]

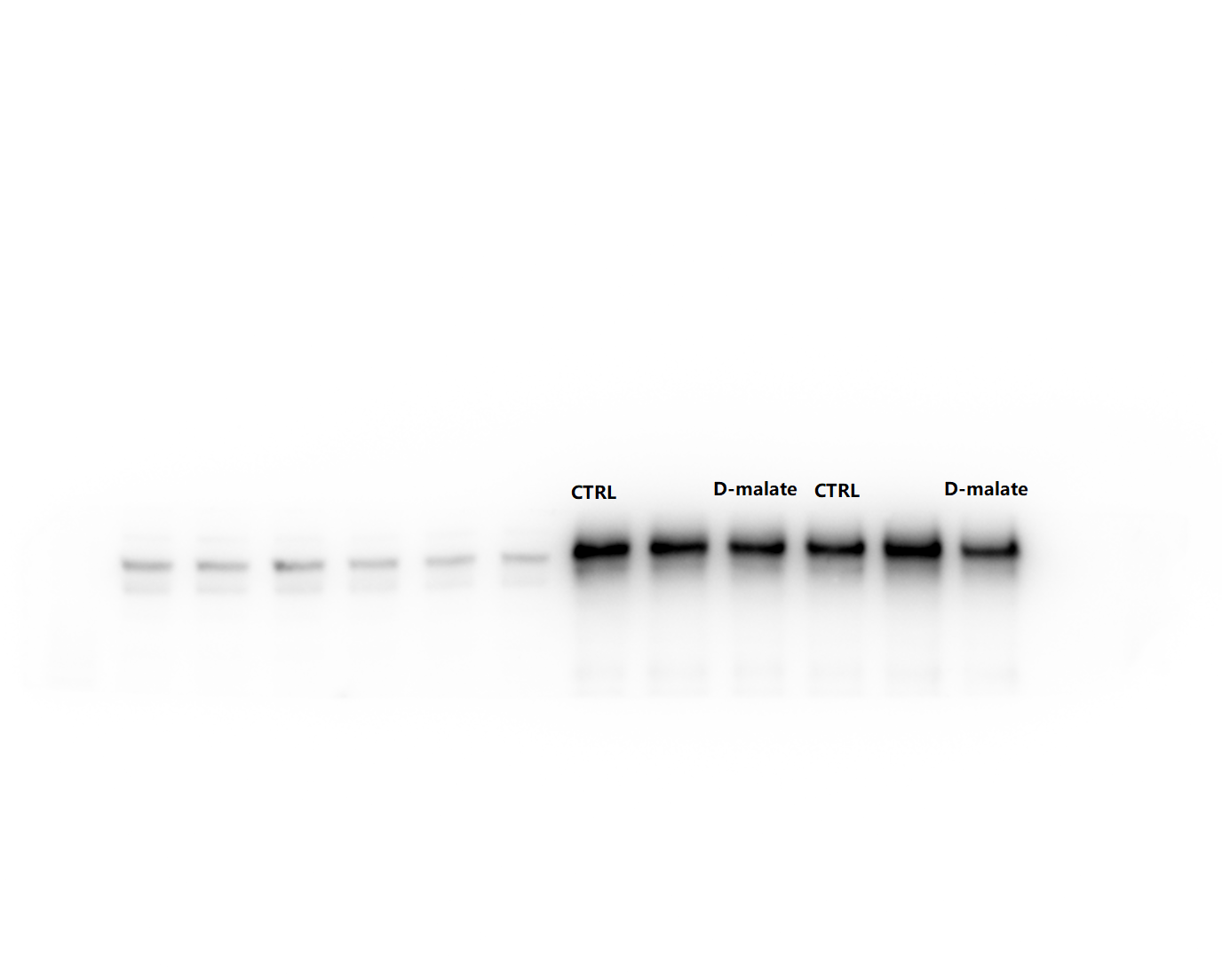

Supplement: Supplementary file 8 — Source Data Fig. 7 [file 44319_2023_28_MOESM8_ESM.zip › EMBOR-2023-57167V1_SourceDataForFigure 7/Fig 7G IP CyclinA.Tif]

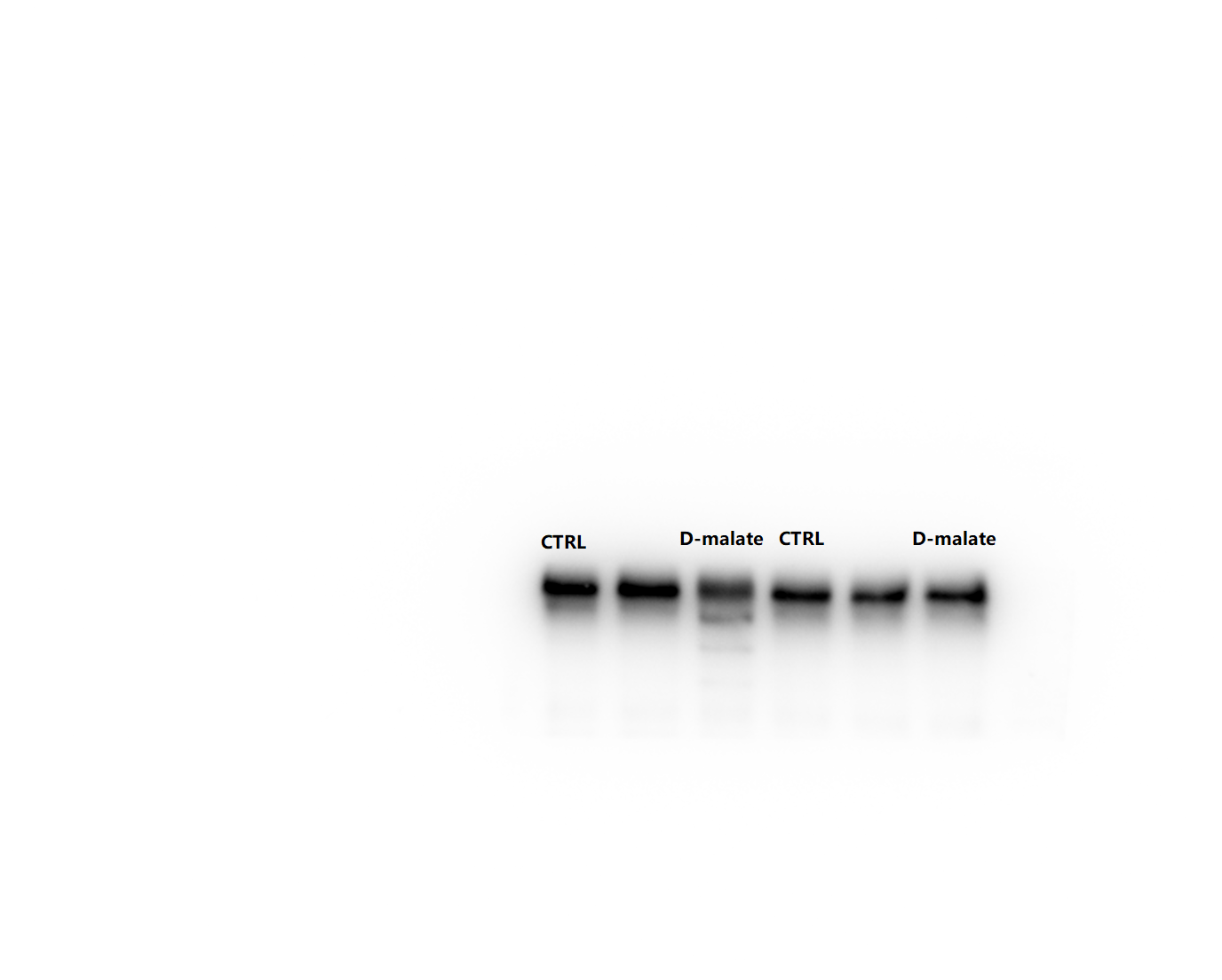

Supplement: Supplementary file 8 — Source Data Fig. 7 [file 44319_2023_28_MOESM8_ESM.zip › EMBOR-2023-57167V1_SourceDataForFigure 7/Fig 7G IP CyclinA(replication).Tif]

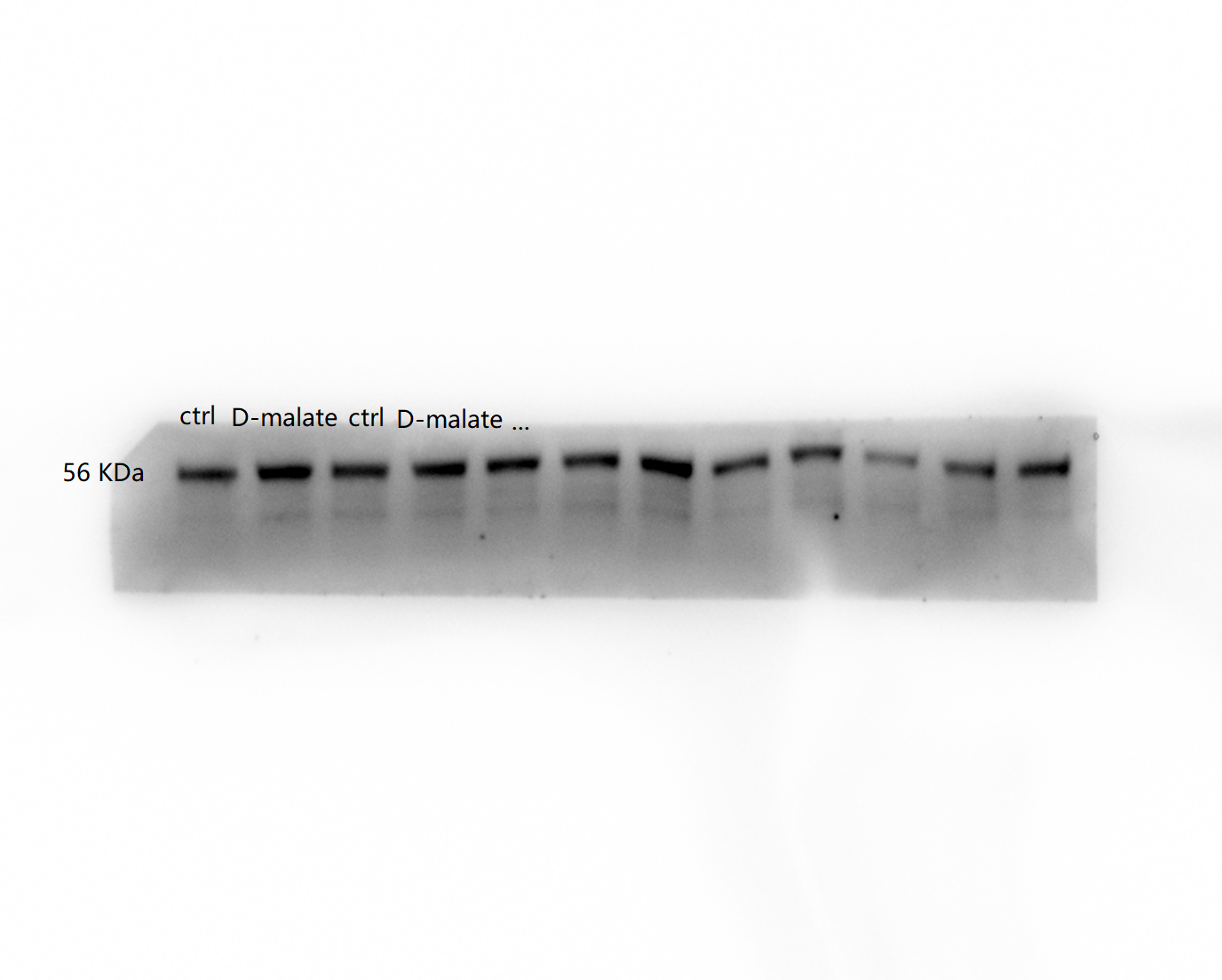

Supplement: Supplementary file 9 — EV Figure Source Data [file 44319_2023_28_MOESM9_ESM.zip › EMBOR-2023-57167V1_SourceDataForExpendedView 1/Fig EV1H-I and Fig EV1L-M/AKT.Tif]

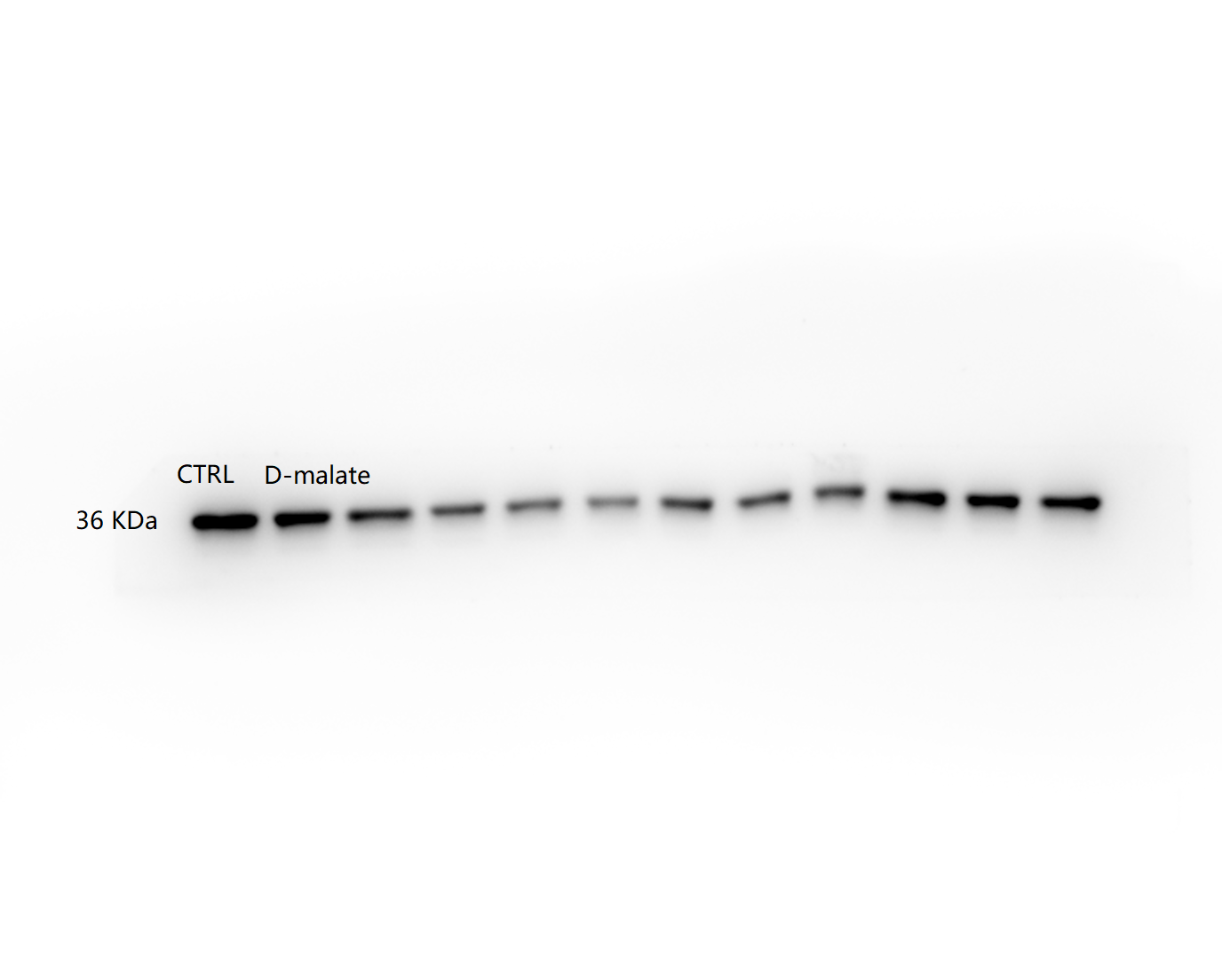

Supplement: Supplementary file 9 — EV Figure Source Data [file 44319_2023_28_MOESM9_ESM.zip › EMBOR-2023-57167V1_SourceDataForExpendedView 1/Fig EV1H-I and Fig EV1L-M/GAPDH.Tif]

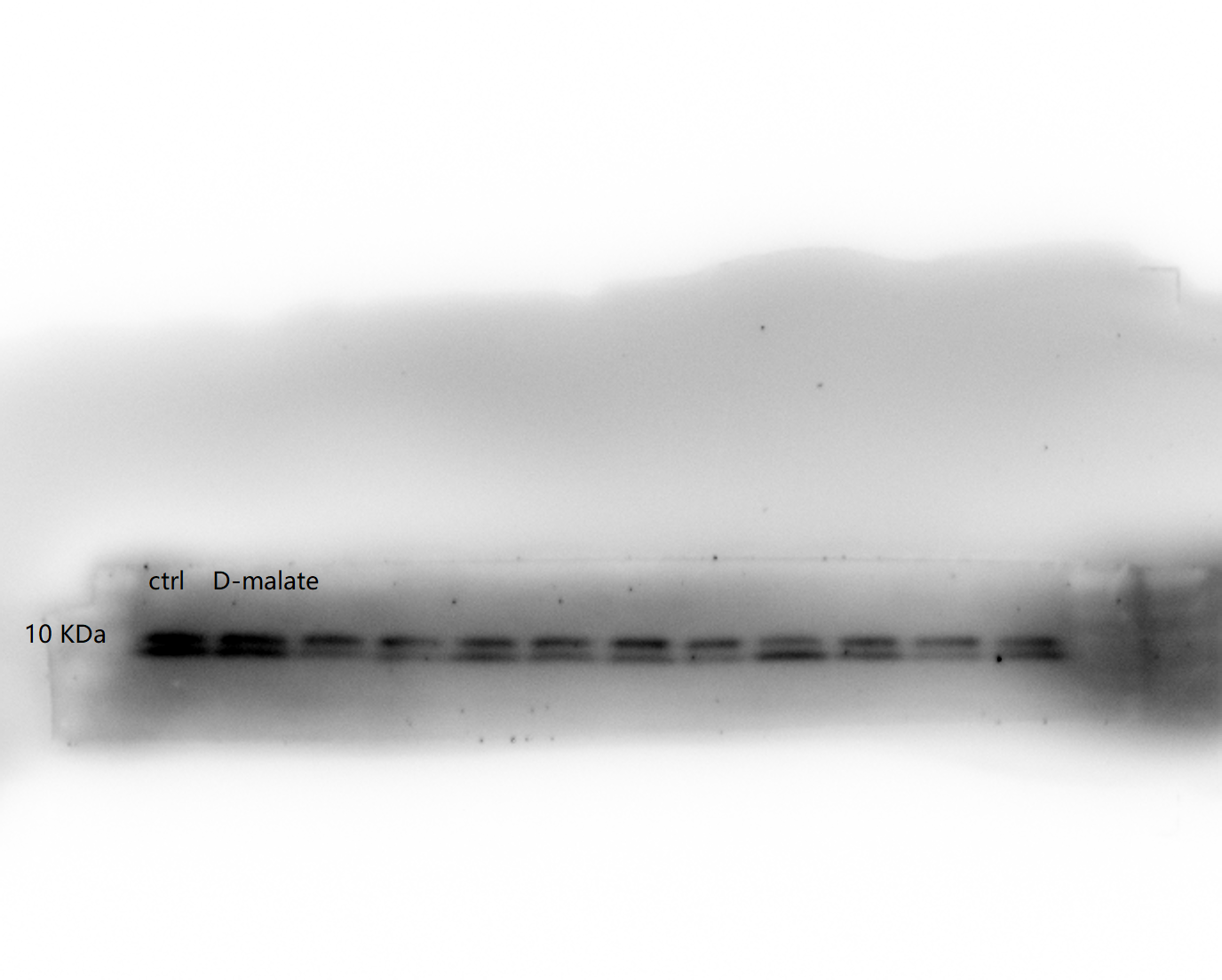

Supplement: Supplementary file 9 — EV Figure Source Data [file 44319_2023_28_MOESM9_ESM.zip › EMBOR-2023-57167V1_SourceDataForExpendedView 1/Fig EV1H-I and Fig EV1L-M/LC3.Tif]

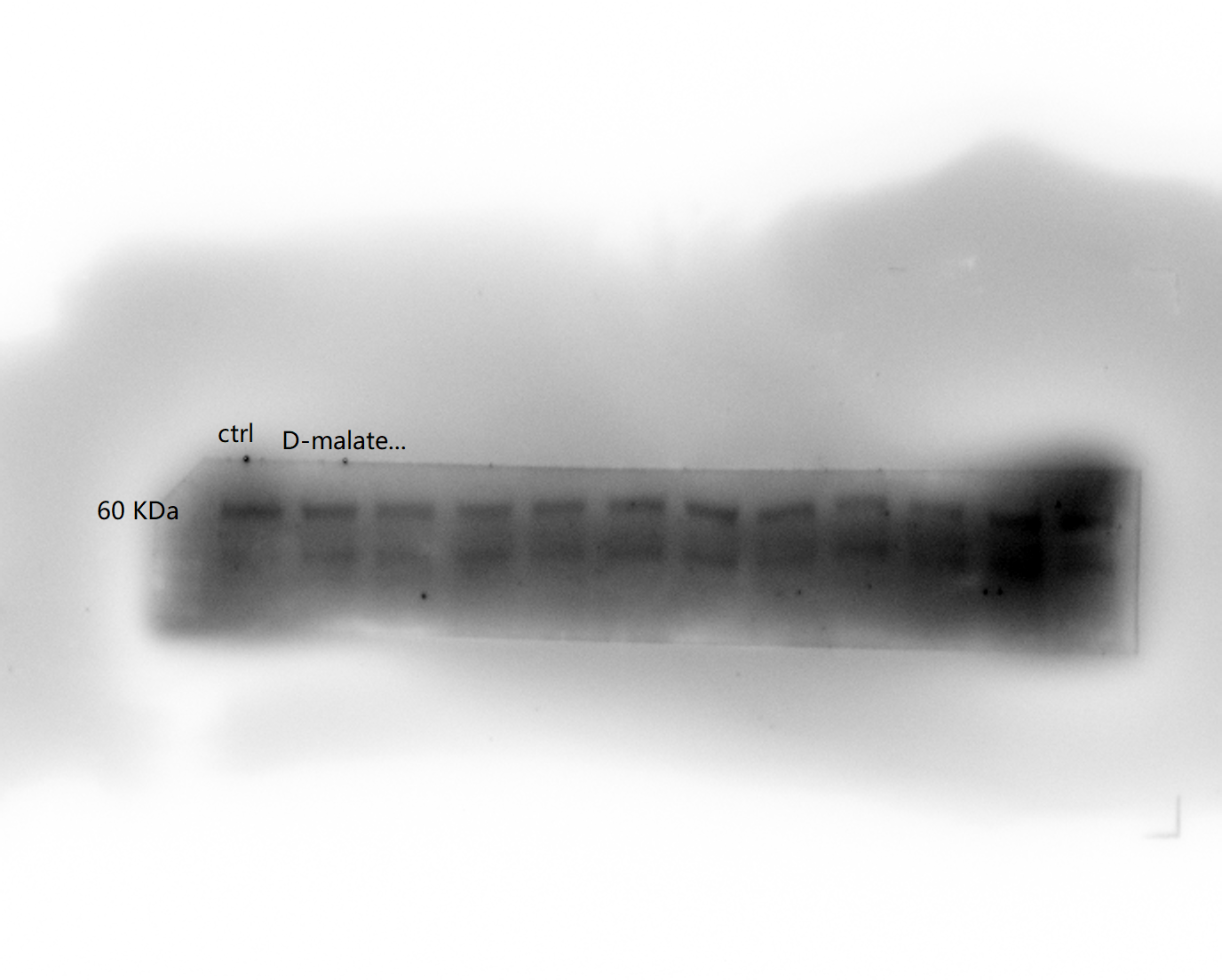

Supplement: Supplementary file 9 — EV Figure Source Data [file 44319_2023_28_MOESM9_ESM.zip › EMBOR-2023-57167V1_SourceDataForExpendedView 1/Fig EV1H-I and Fig EV1L-M/P-AKT.Tif]

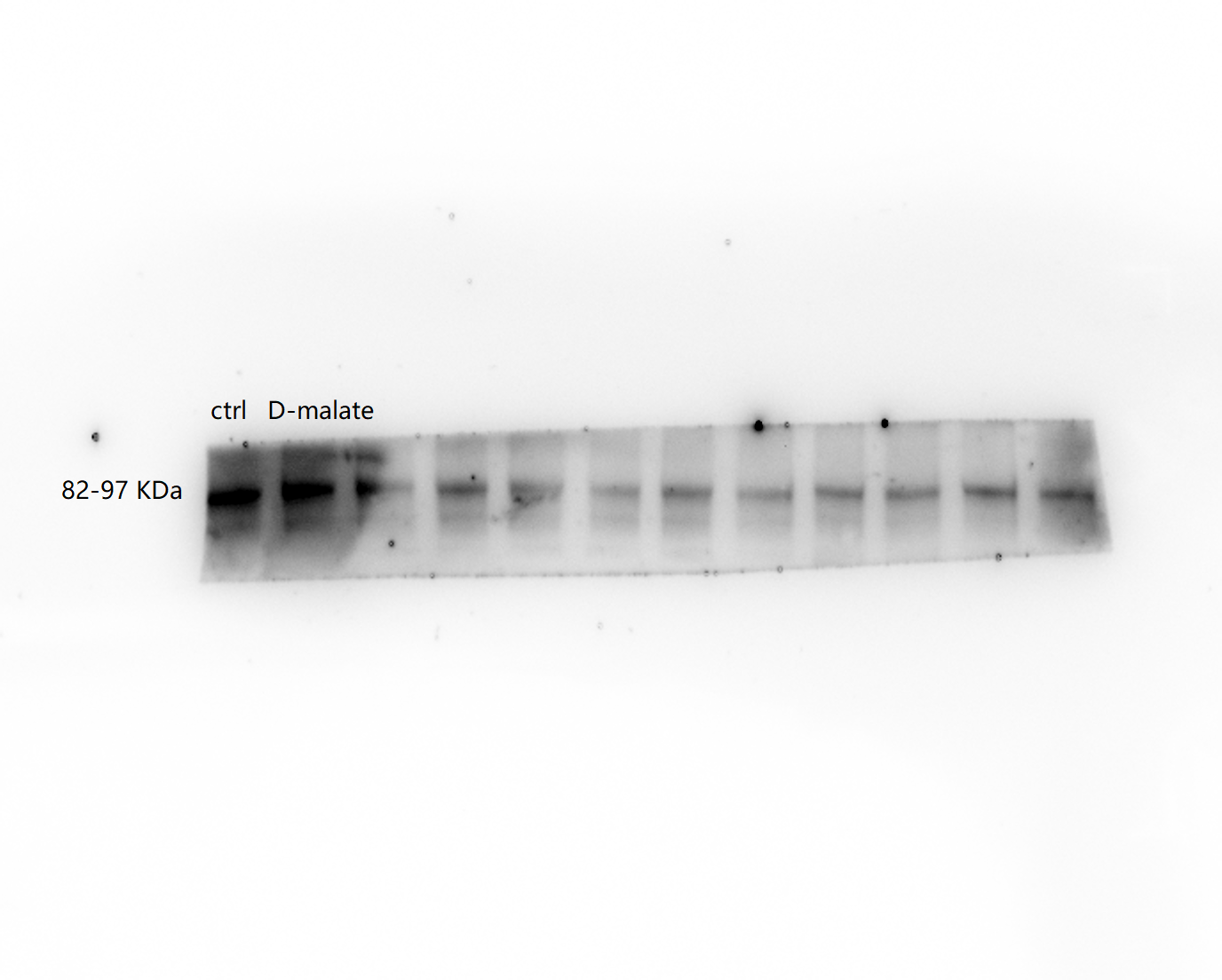

Supplement: Supplementary file 9 — EV Figure Source Data [file 44319_2023_28_MOESM9_ESM.zip › EMBOR-2023-57167V1_SourceDataForExpendedView 1/Fig EV1J-K/FOXO3.Tif]

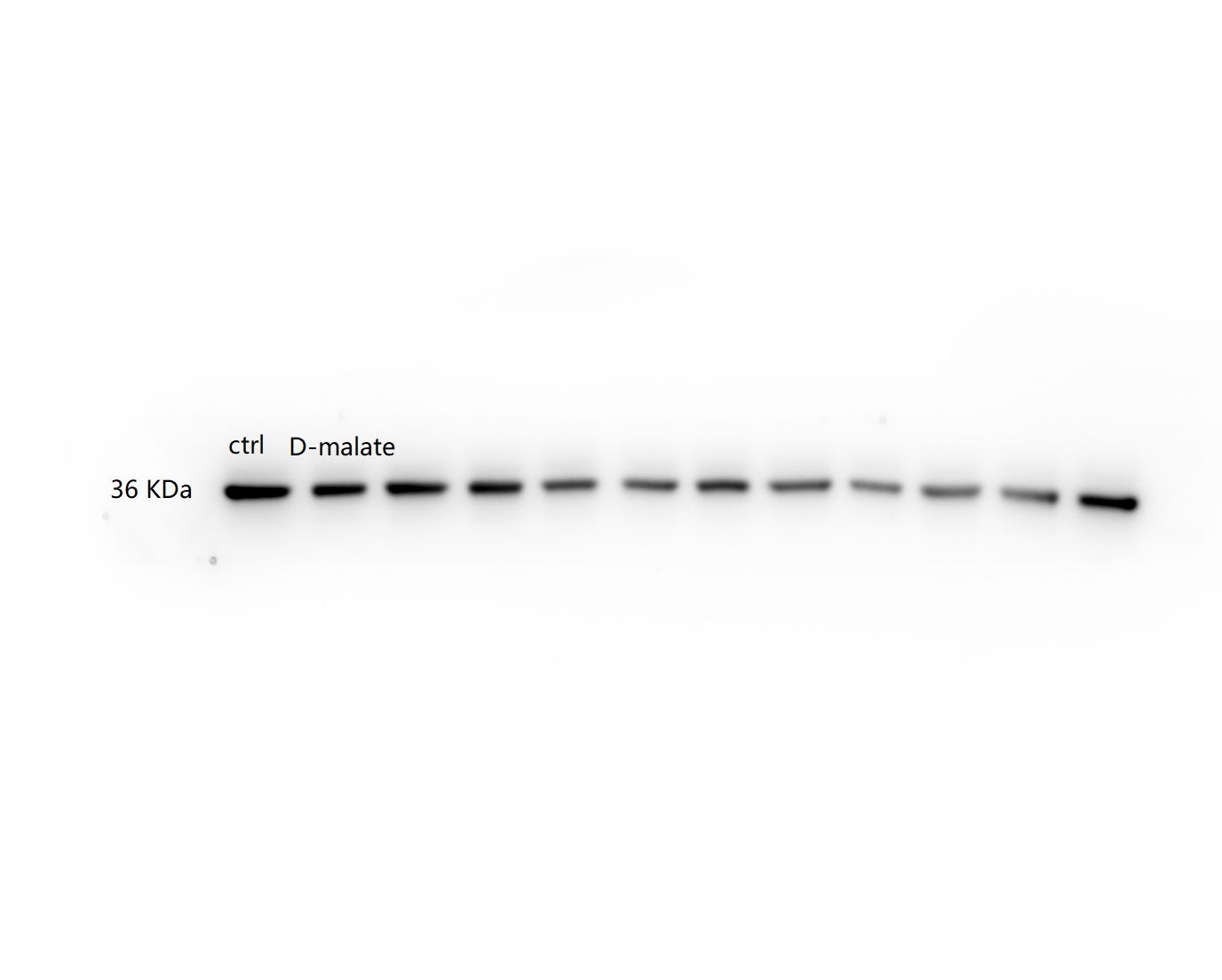

Supplement: Supplementary file 9 — EV Figure Source Data [file 44319_2023_28_MOESM9_ESM.zip › EMBOR-2023-57167V1_SourceDataForExpendedView 1/Fig EV1J-K/GAPDH.Tif]

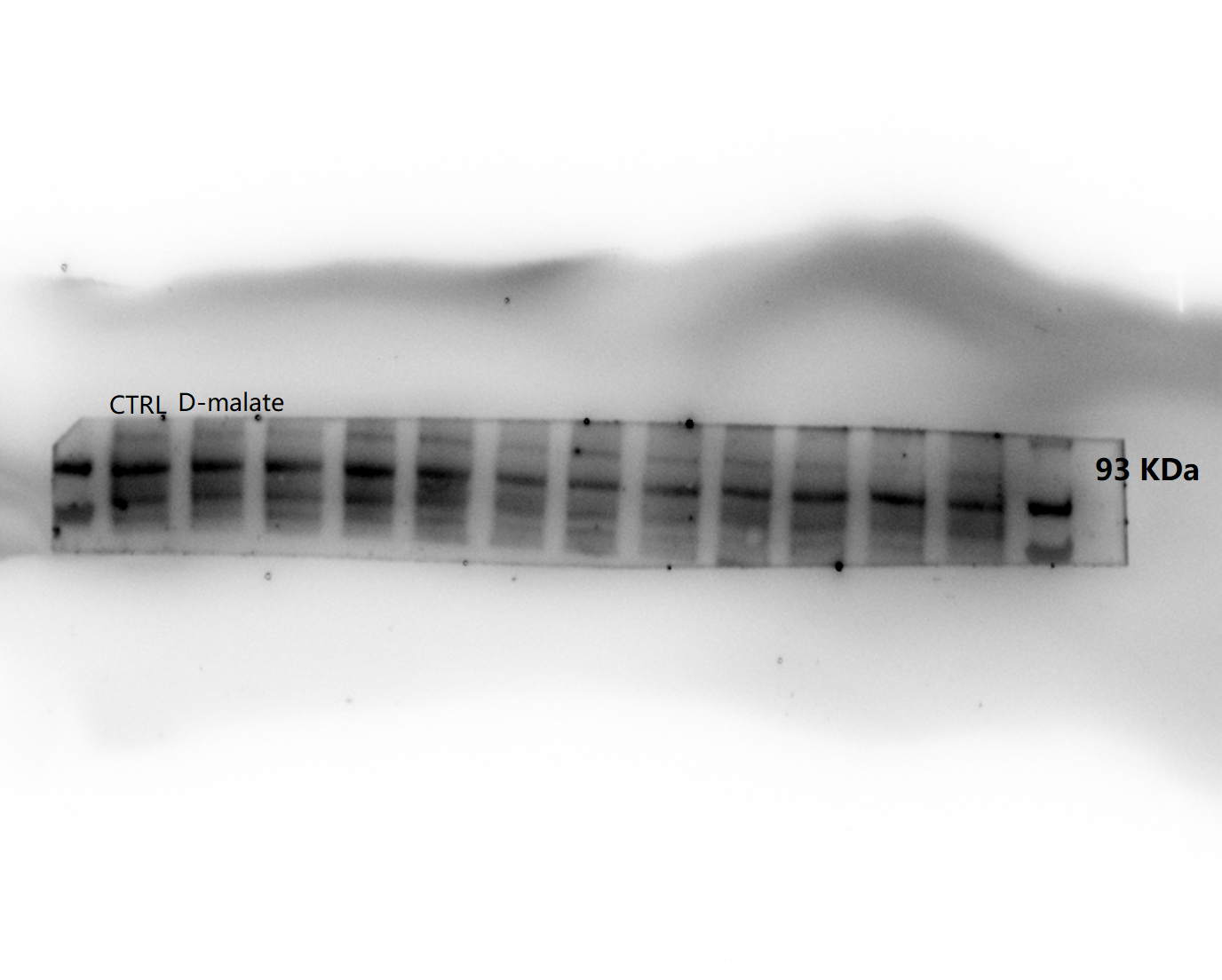

Supplement: Supplementary file 9 — EV Figure Source Data [file 44319_2023_28_MOESM9_ESM.zip › EMBOR-2023-57167V1_SourceDataForExpendedView 1/Fig EV1J-K/P-FOXO3.Tif]

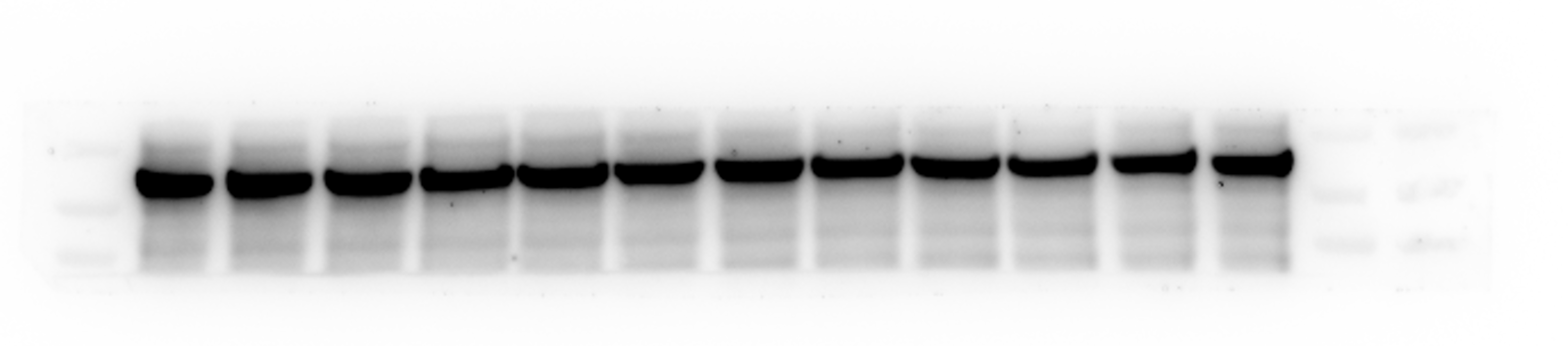

Supplement: Supplementary file 9 — EV Figure Source Data [file 44319_2023_28_MOESM9_ESM.zip › EMBOR-2023-57167V1_SourceDataForExpendedView 2/Fig EV2F Actin.tif]

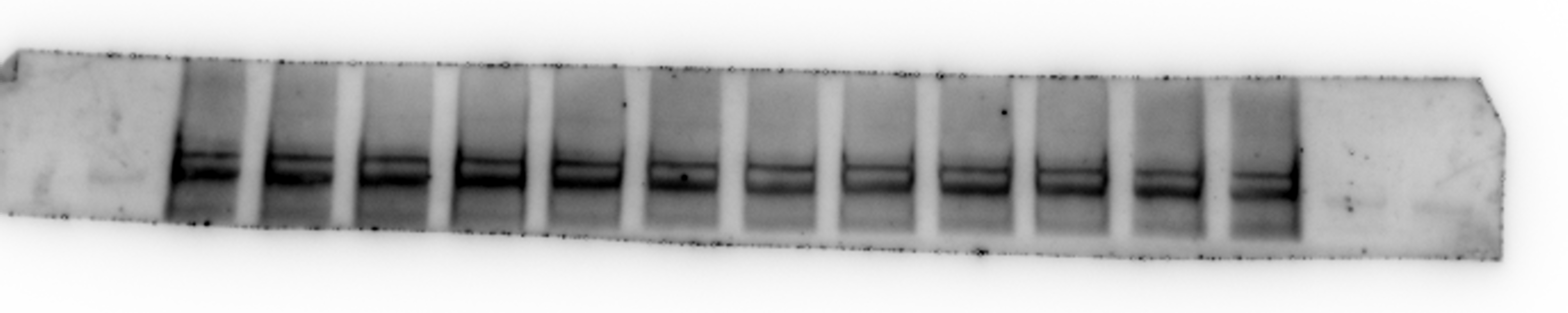

Supplement: Supplementary file 9 — EV Figure Source Data [file 44319_2023_28_MOESM9_ESM.zip › EMBOR-2023-57167V1_SourceDataForExpendedView 2/Fig EV2F mTOR.tif]

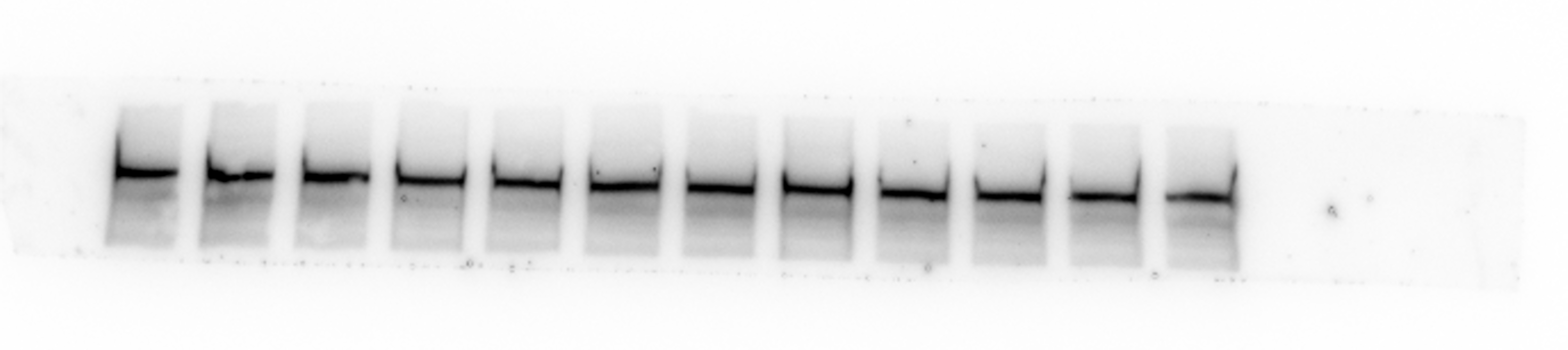

Supplement: Supplementary file 9 — EV Figure Source Data [file 44319_2023_28_MOESM9_ESM.zip › EMBOR-2023-57167V1_SourceDataForExpendedView 2/Fig EV2F P-mTOR.tif]

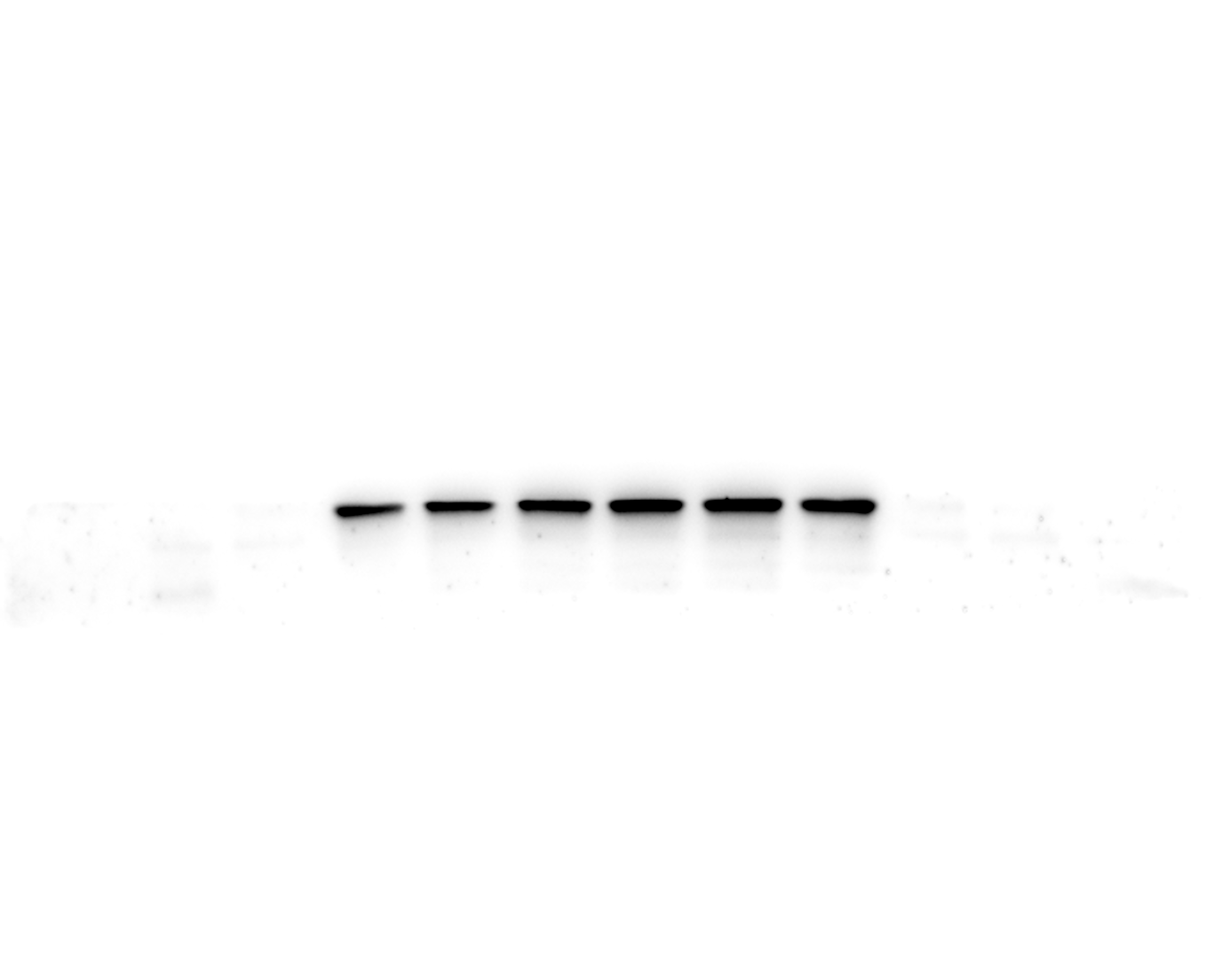

Supplement: Supplementary file 9 — EV Figure Source Data [file 44319_2023_28_MOESM9_ESM.zip › EMBOR-2023-57167V1_SourceDataForExpendedView 2/Fig EV2H GAPDH.Tif]

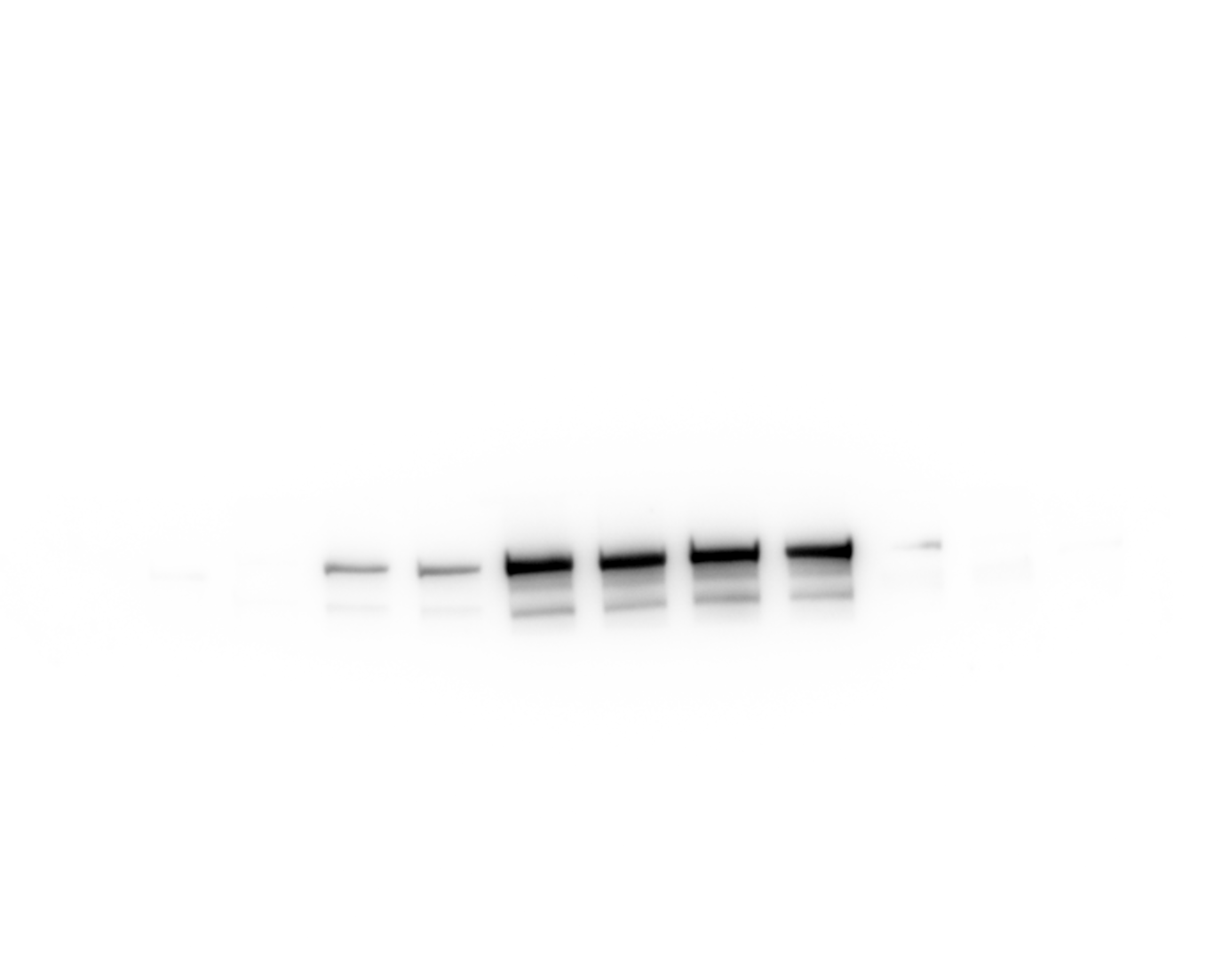

Supplement: Supplementary file 9 — EV Figure Source Data [file 44319_2023_28_MOESM9_ESM.zip › EMBOR-2023-57167V1_SourceDataForExpendedView 2/Fig EV2H MYHC.Tif]

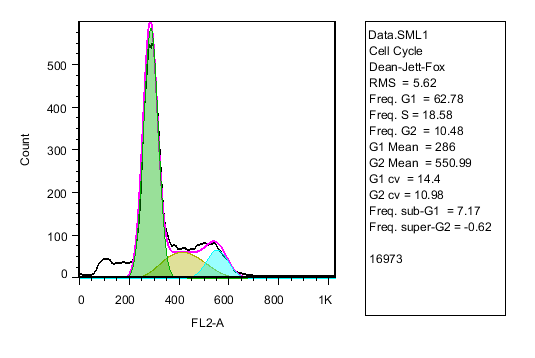

Supplement: Supplementary file 9 — EV Figure Source Data [file 44319_2023_28_MOESM9_ESM.zip › EMBOR-2023-57167V1_SourceDataForExpendedView 3/Fig EV3A-B souce data/SML1.png]

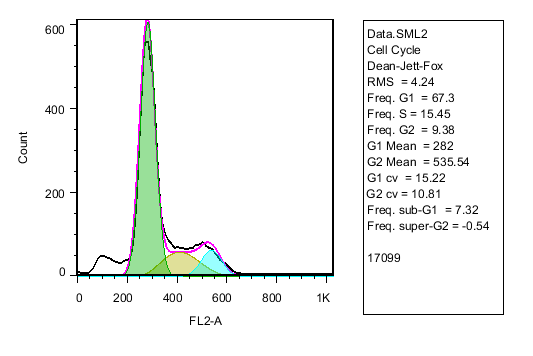

Supplement: Supplementary file 9 — EV Figure Source Data [file 44319_2023_28_MOESM9_ESM.zip › EMBOR-2023-57167V1_SourceDataForExpendedView 3/Fig EV3A-B souce data/SML2.png]

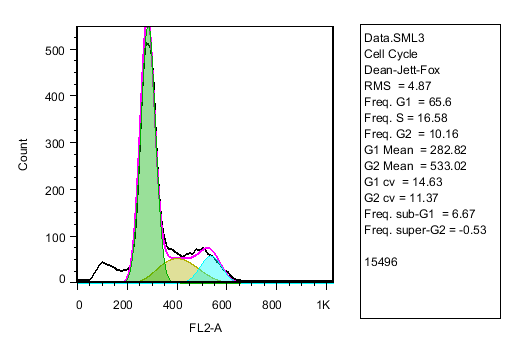

Supplement: Supplementary file 9 — EV Figure Source Data [file 44319_2023_28_MOESM9_ESM.zip › EMBOR-2023-57167V1_SourceDataForExpendedView 3/Fig EV3A-B souce data/SML3.png]

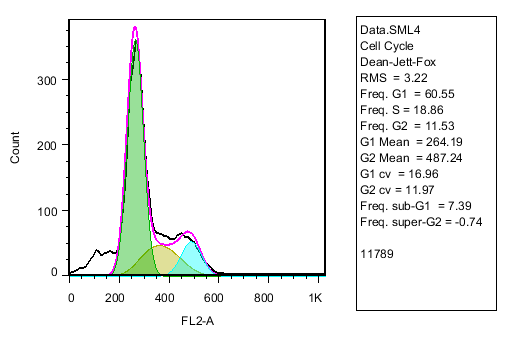

Supplement: Supplementary file 9 — EV Figure Source Data [file 44319_2023_28_MOESM9_ESM.zip › EMBOR-2023-57167V1_SourceDataForExpendedView 3/Fig EV3A-B souce data/SML4.png]

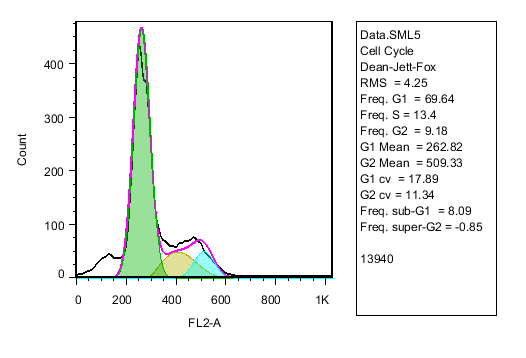

Supplement: Supplementary file 9 — EV Figure Source Data [file 44319_2023_28_MOESM9_ESM.zip › EMBOR-2023-57167V1_SourceDataForExpendedView 3/Fig EV3A-B souce data/SML5.png]

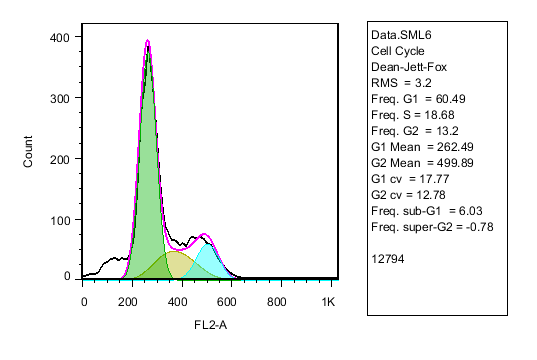

Supplement: Supplementary file 9 — EV Figure Source Data [file 44319_2023_28_MOESM9_ESM.zip › EMBOR-2023-57167V1_SourceDataForExpendedView 3/Fig EV3A-B souce data/SML6.png]

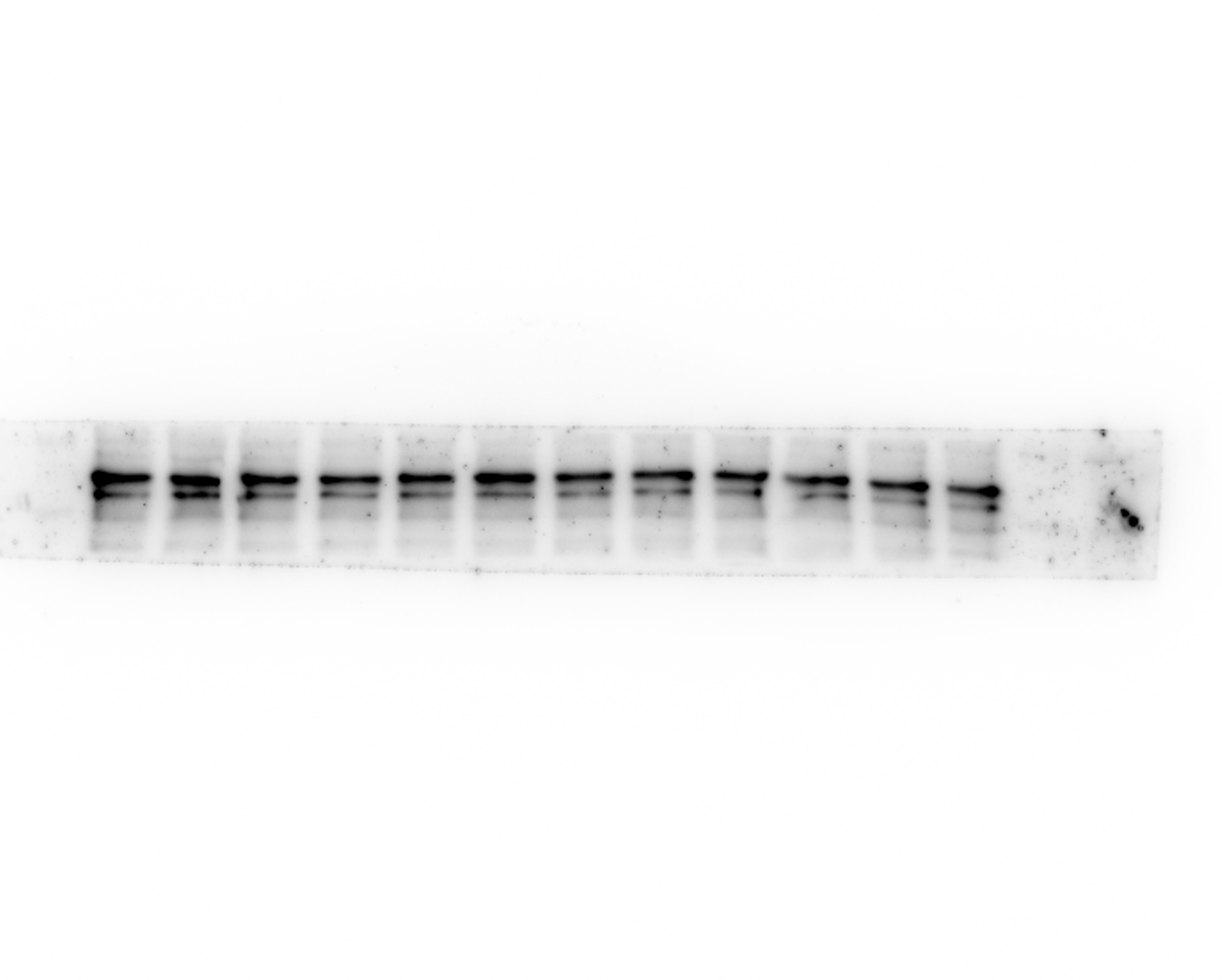

Supplement: Supplementary file 9 — EV Figure Source Data [file 44319_2023_28_MOESM9_ESM.zip › EMBOR-2023-57167V1_SourceDataForExpendedView 3/Fig EV3C GAPDH.Tif]

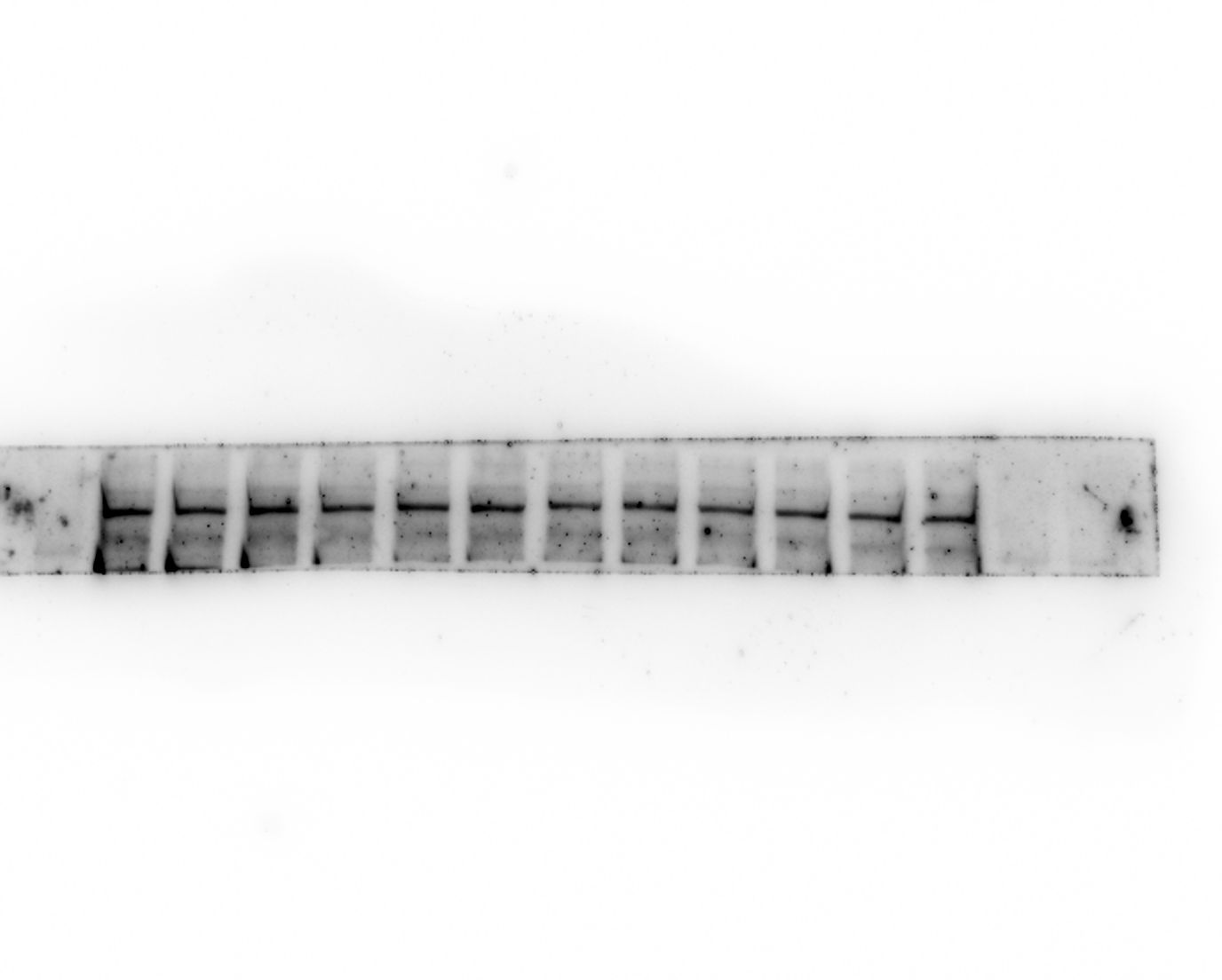

Supplement: Supplementary file 9 — EV Figure Source Data [file 44319_2023_28_MOESM9_ESM.zip › EMBOR-2023-57167V1_SourceDataForExpendedView 3/Fig EV3C MLCK.Tif]
