# Supplementary material for: Microbiota derived d-malate inhibits skeletal muscle growth and angiogenesis during aging via acetylation of Cyclin A
Source: EMBO Rep. 2024 Jan 22;25(2):9. doi: 10.1038/s44319-023-00028-y (PMC10897302; doi:10.1038/s44319-023-00028-y)
Supplement: Supplementary file 10 — Expanded View Figures [file 44319_2023_28_MOESM10_ESM.pdf]

Expanded View Figures

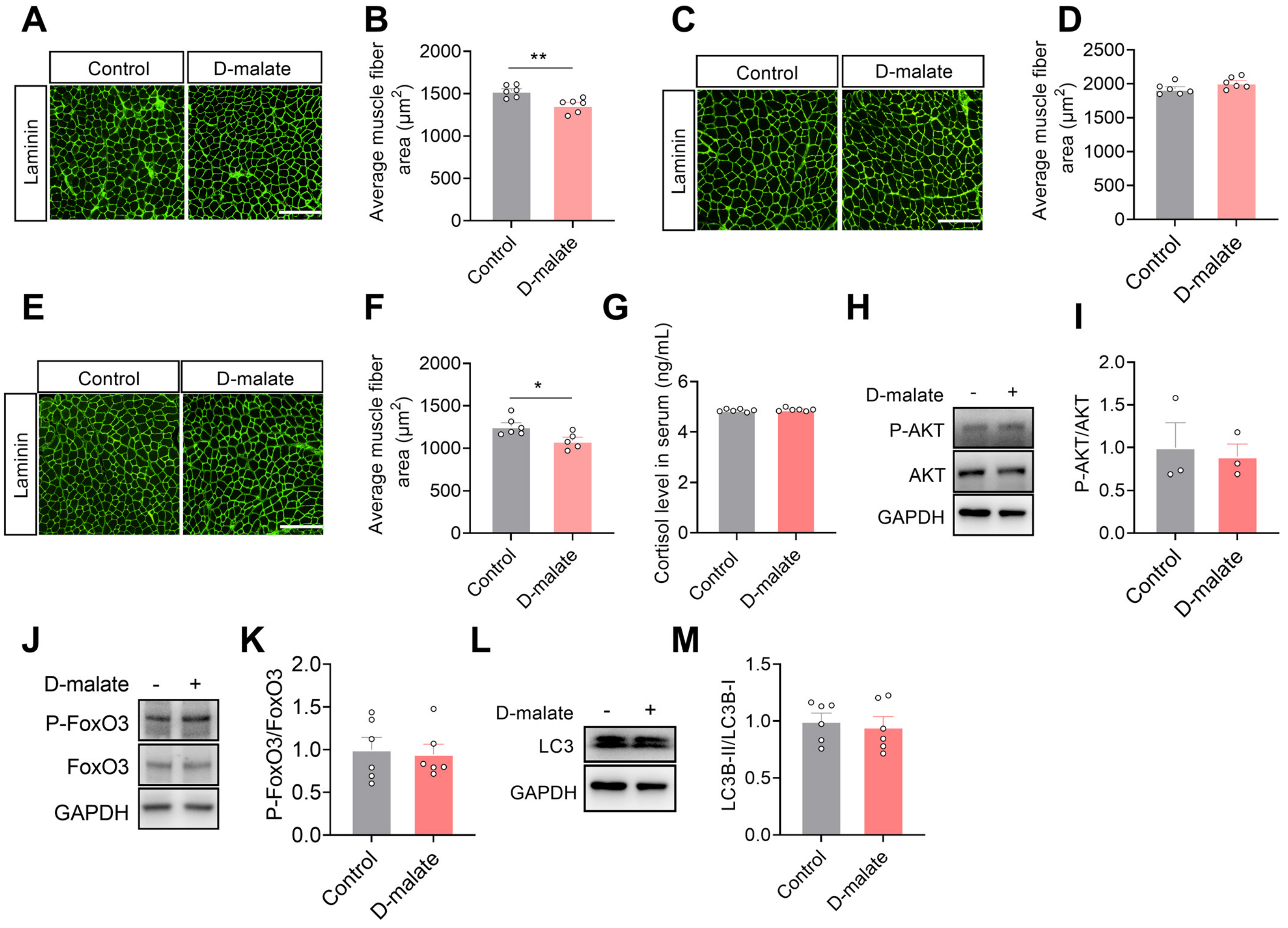

Figure EV1. The effects of D-malate in CSA of TA/SOL/EDL and protein turnover.

(**A–F**) The laminin immunofluorescent staining and frequency histogram of muscle fiber cross-sectional area statistical analysis in tibialis anterior (**A**, **B**), solus (**C**, **D**), and extensor digitorum longus (**E**, **F**) ($n = 6$). Scale bars, 200 μm. (**G**) Serum cortisol level of 12-week-old male mice with 6-week D-malate treatment ($n = 6$). (**H–M**) The protein expression of P-AKT (**H**, **I**), P-FoxO3 (**J**, **K**) and LC3 (**L**, **M**) in gastrocnemius ($n = 6$). Data information: *t* test was used in this figure where error bars represent SEM, and $^{*}P < 0.05$; $^{**}P < 0.01$. Source data are available online for this figure.

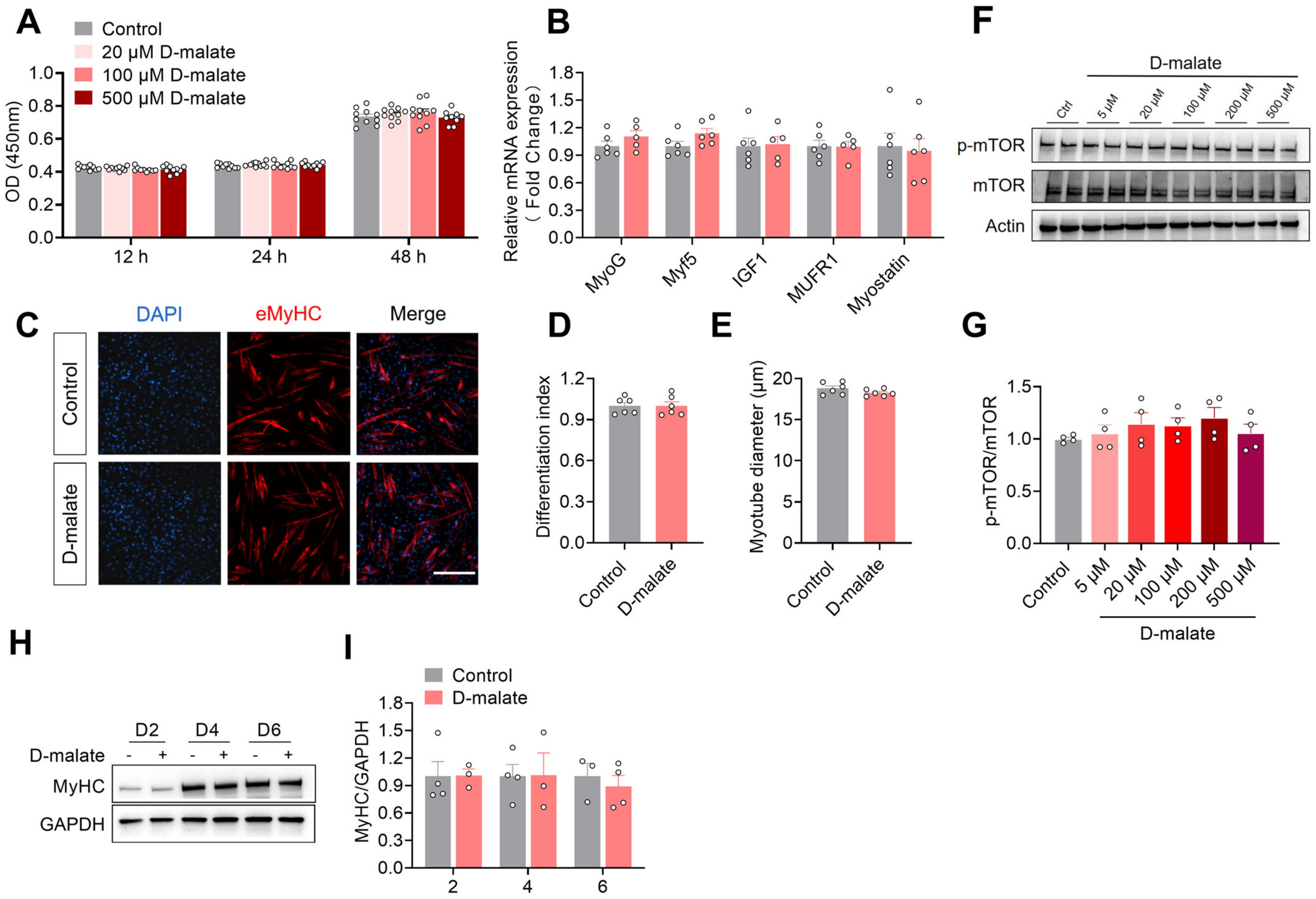

Figure EV2. D-malate had no function in the proliferation, differentiation and P-mTOR of C2C12.

(**A**) OD value of CCK-8 to detect proliferation activity of C2C12 cells ($n = 10$). (**B**) The mRNA expression of MyoG, Myf5, IGF1, MUFR1, Myostatin in C2C12 cells ($n = 5$–6). (**C–E**) Immunofluorescence images (**C**) of C2C12 cells after induced differentiation, differentiation index (**D**) and myotube diameter (**E**) ($n = 6$). Scale bars, 200 μm. (**F, G**) The protein expression of p-mTOR/mTOR in C2C12 cells ($n = 4$). (**H, I**) The protein expression of MyHC in C2C12 cells with 100 μM D-malate treatment for 2 days, 4 days and 6 days, ctrl group didn't receive D-malate treatment. ($n = 3$). Data information: *t* test was used in this figure where error bars represent SEM. Source data are available online for this figure.

 © The Author(s)

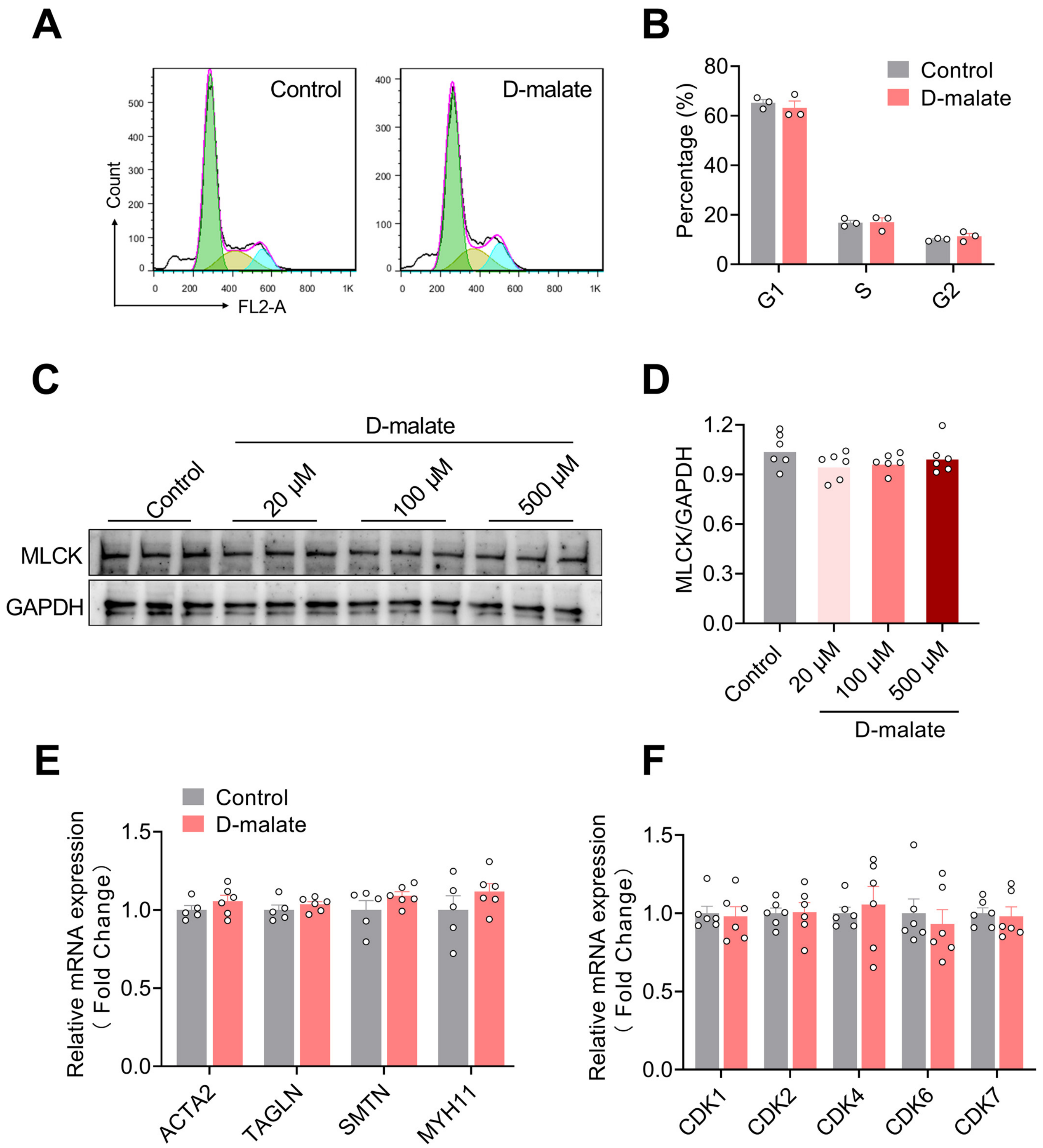

Figure EV3. D-malate had no function in cell cycle and vasomotor function of vascular smooth muscle cell.

(**A**, **B**) Representative image (**A**) and statistical graph (**B**) of vascular smooth muscle cell cycle by flow cytometry ($n = 5$–6). (**C**, **D**) The protein expression of MLCK in vascular smooth muscle cells ($n = 6$). (**E**) The mRNA expression of ACTA2, TAGLN, SMTN and MYH11 in vascular smooth muscle cells ($n = 5$–6). (**F**) The mRNA expression of cyclin-dependent kinases in vascular endothelial cells ($n = 6$). Data information: *t* test was used in this figure where error bars represent SEM. Source data are available online for this figure.

© The Author(s)

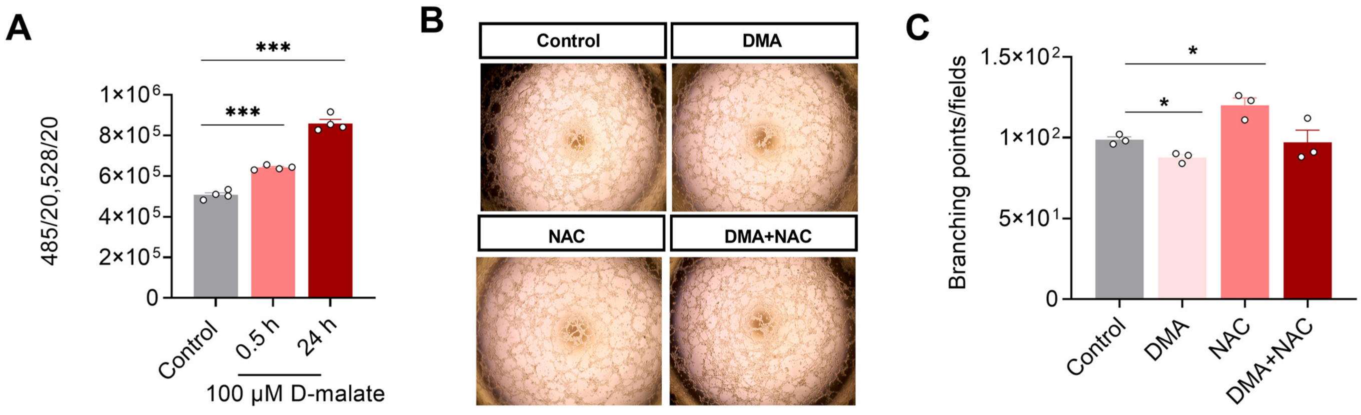

Figure EV4. D-malate inhibits vascular endothelial cell proliferation is independent of ROS.

(**A**) The ROS content in vascular endothelial cell was detected by ROS kit ($n = 4$). (**B**, **C**) Representative images (**B**) and statistics (**C**) of vascular endothelial cell tube formation test within NAC and D-malate treatment ($n = 3$). Data information: *t* test was used in this figure where error bars represent SEM, and $^{*}P < 0.05$; $^{***}P < 0.001$. Source data are available online for this figure.

 © The Author(s)
